# Synthetic biology open language visual (SBOL visual) version 3.0

**DOI:** 10.1515/jib-2021-0013

**Published:** 2021-10-20

**Authors:** Hasan Baig, Pedro Fontanarossa, James McLaughlin, James Scott-Brown, Prashant Vaidyanathan, Thomas Gorochowski, Goksel Misirli, Jacob Beal, Chris Myers

**Affiliations:** University of Connecticut, Storrs, USA; University of Utah, Salt Lake City, USA; EMBL-EBI, Hinxton, UK; University of Oxford, Oxford, UK; Microsoft Research, Cambridge, UK; University of Bristol, Bristol, UK; Keele University, Newcastle, UK; Raytheon BBN Technologies, Cambridge, USA; University of Colorado Boulder, Boulder, USA

**Keywords:** diagrams, SBOL visual, standards

## Abstract

People who engineer biological organisms often find it useful to draw diagrams in order to communicate both the structure of the nucleic acid sequences that they are engineering and the functional relationships between sequence features and other molecular species. Some typical practices and conventions have begun to emerge for such diagrams. SBOL Visual aims to organize and systematize such conventions in order to produce a coherent language for expressing the structure and function of genetic designs. This document details version 3.0 of SBOL Visual, a new major revision of the standard. The major difference between SBOL Visual 3 and SBOL Visual 2 is that diagrams and glyphs are defined with respect to the SBOL 3 data model rather than the SBOL 2 data model. A byproduct of this change is that the use of dashed undirected lines for subsystem mappings has been removed, pending future determination on how to represent general SBOL 3 constraints; in the interim, this annotation can still be used as an annotation. Finally, deprecated material has been removed from collection of glyphs: the deprecated “insulator” glyph and “macromolecule” alternative glyphs have been removed, as have the deprecated BioPAX alternatives to SBO terms.

**Author contribution:** All authors have accepted responsibility for the entire content of this manuscript and approved its submission.
**Research funding:** The results of this work was funded by grant National Science Foundation.
**Conflict of interest statement:** Authors state no conflict of interest.

## Synthetic Biology Open Language Visual (SBOL Visual) Version 3.0

**Editors:**

Hasan Baig — *University of Connecticut, USA*
Pedro Fontanarrosa — *University of Utah, USA*
James McLaughlin — *EMBL-EBI, UK*
James Scott-Brown — *University of Oxford, UK*
Prashant Vaidyanathan — *Microsoft Research, UK*

sbol-editors@googlegroups.com

**Chair:**

Chris Myers — *University of Colorado Boulder, USA*

**Additional authors, by last name:**

Jacob Beal — *Raytheon BBN Technologies, USA*
Thomas Gorochowski — *University of Bristol, UK*
Goksel Misirli — *Keele University, UK*

Version 3.0

2021-04-14

### Contents

1 Purpose 3
1.1 Relation to Data Models 3
2 Relation to other Standards 3
3 SBOL Specification Vocabulary 4
3.1 Term Conventions 4
3.2 SBOL Class Names 4
4 SBOL Glyphs 6
4.1 Requirements for Glyphs 7
4.2 Reserved Visual Properties 8
4.3 Extending the Set of Glyphs 8
5 SBOL Visual Diagram Language 10
5.1 Nucleic Acid Backbone 10
5.2 Sequence Features 13
5.3 Molecular Species 15
5.4 Interactions 15
5.5 Subsystems 17
5.6 Labels 19
5.7 Annotations 19
5.8 Criteria for Compliance with SBOL Visual 20
A SBOL Visual Glyphs 21
A.1 Sequence Feature Glyphs 21
A.2 Molecular Species Glyphs 62
A.3 Interaction Glyphs 72
A.4 Interaction Node Glyphs 78
B Examples 84
C Mapping between to SBOL Visual 1, 2, 3 89
C.1 Mapping between SBOL Visual 1 and SBOL Visual 2 89
C.2 Mapping between SBOL Visual 2 and SBOL Visual 3 90
References 91

## 1 Purpose

People who engineer biological organisms often find it useful to draw diagrams in order to communicate both the structure of the nucleic acid sequences that they are engineering and the functional relationships between sequence features and other molecular species. Some typical practices and conventions have begun to emerge for such diagrams. SBOL Visual aims to organize and systematize such conventions in order to produce a coherent language for expressing the structure and function of genetic designs. At the same time, we aim to make this language simple and easy to use, allowing a high degree of flexibility and freedom in how such diagrams are organized, presented, and styled—in particular, it should be readily possible to create diagrams either by hand or using a wide variety of software programs. Finally, means are provided for extending the language with new and custom diagram elements, and for adoption of useful new elements into the language.

### 1.1 Relation to Data Models

In order to ground SBOL Visual with precise definitions, we reference its visual elements to data models with well-defined semantics. In particular, glyphs and diagrams in SBOL Visual are defined in terms of their relation to the SBOL 3 data model (Baig et al., 2020) and terms in the Sequence Ontology (Eilbeck et al., 2005) and the Systems Biology Ontology (Courtot et al., 2011).

SBOL Visual is not intended to represent designs at the same level of detail as these data models. Effective visual diagrams are necessarily more abstract, focusing only on those aspects of a system that are the subject of the communication. Nevertheless, we take as a principle that it should be possible to transform any SBOL Visual diagram into an equivalent (if highly abstract) SBOL 3 data representation. Likewise, we require that SBOL Visual should be able to represent all of the significant structural or functional relationships in any GenBank or SBOL data representation.

## 2 Relation to other Standards

SBOL Visual 3.0 replaces SBOL Visual 2.3.

SBOL Visual 2.3 also implicitly supersedes the previously replaced SBOL Visual 2.2 and 2.1, as well as BBF RFC 115 (SBOL Visual 2.0), BBF RFC 93 (SBOL Visual 1.1) and BBF RFC 16 (SBOL Visual 1.0).

Every glyph in SBOL Visual 3.0 corresponds to an element of the SBOL 3.0 data model (Baig et al., 2020). SBOL Visual 3.0 also defines many terms by reference to SBOL 3.0, or by reference to the Sequence Ontology (Eilbeck et al., 2005) or the Systems Biology Ontology (Courtot et al., 2011).

SBOL Visual is intended to be compatible with the Systems Biology Graphical Notation Activity Flow Language (SBGN AF) (Le Novère et al., 2009), and species and interaction glyphs have been imported from that language (see: Appendix A.2 and Appendix A.3). Some aspects are also imported from the Systems Biology Graphical Notation Process Description Language (SBGN PD).

## 3 SBOL Specification Vocabulary

### 3.1 Term Conventions

This document indicates requirement levels using the controlled vocabulary specified in IETF RFC 2119 and reiterated in BBF RFC 0. In particular, the key words "MUST", "MUST NOT", "REQUIRED", "SHALL", "SHALL NOT", "SHOULD", "SHOULD NOT", "RECOMMENDED", "MAY", and "OPTIONAL" in this document are to be interpreted as described in RFC 2119:

- The words "MUST", "REQUIRED", or "SHALL" mean that the item is an absolute requirement of the specification.
- The phrases "MUST NOT" or "SHALL NOT" mean that the item is an absolute prohibition of the specification.
- The word "SHOULD" or the adjective "RECOMMENDED" mean that there might exist valid reasons in particular circumstances to ignore a particular item, but the full implications need to be understood and carefully weighed before choosing a different course.
- The phrases "SHOULD NOT" or "NOT RECOMMENDED" mean that there might exist valid reasons in particular circumstances when the particular behavior is acceptable or even useful, but the full implications need to be understood and the case carefully weighed before implementing any behavior described with this label.
- The word "MAY" or the adjective "OPTIONAL" mean that an item is truly optional.

### 3.2 SBOL Class Names

The definition of SBOL Visual references several SBOL classes, which are defined as listed here. For full definitions and explanations, see the SBOL 3.0 data model (Baig et al., 2020).

- *`Component`*: Describes the structure of designed entities, such as DNA, RNA, and proteins, as well as other entities they interact with, such as small molecules or environmental properties, and the functional relationships and constraints relating these elements.
- *`Feature`*: Represents a specific occurrence or instance of an entity within the design of a `Component`, such as a promoter in a genetic construct or an enzyme within a synthesis reaction network.
- *`SubComponent`*: A type of `Feature` that indicates the inclusion of a potentially complicated sub-construct or sub-system within the design of a `Component`. For example, a `SubComponent` might represent one gene (with all its attendant complexity) within a multi-gene design or might represent a set of linked reactions within a larger metabolic network.
- *`ComponentReference`*: A type of `Feature` that is defined by reference to another `Feature` in a `SubComponent`. For example, a `ComponentReference` might be used to indicate a regulation of the promoter in a functional component design included as a `SubComponent` or a reaction involving the product of a reaction network included as a `SubComponent`.
- *`Location`*: Specifies the base coordinates and orientation of a genetic feature on a DNA or RNA molecule or a residue or site on another sequential macromolecule such as a protein.
- *`Constraint`*: Describes the relative spatial position, sequence orientation, topological relationship, or identity relationship of two `Feature` objects that are contained within the same `Component`.
- *`Interaction`*: Describes a functional relationship between `Feature` objects, such as regulatory activation or repression, or a biological process such as transcription or translation.

- *Participation*: Describes the role that a Feature plays in an Interaction. For example, a transcription factor might participate in an Interaction as a repressor or as an activator.
- *Interface*: Describes the intended interface for a Component by designating a set of Feature objects as input, output, or nondirectional elements of the interface.

## 4 SBOL Glyphs

A glyph is a visual symbol used to represent an element in an SBOL Visual diagram. All of the currently defined glyphs are collected in Appendix A. This section explains how glyphs are specified and how to add new glyphs.

Each SBOL glyph is defined by association with ontology terms, and can be used to represent any diagram element that is well-described by that term. Currently there are four classes of glyphs, each associated with an ontology and a class in the SBOL 3 data model:

- **Sequence Feature Glyphs** describe features of nucleic acid sequences. They are associated with Sequence Ontology terms. For the SBOL 3 data model, this is formally defined as any `Feature` with a compatible term within its associated `roles`, i.e., one that is equal to or a child of at least one term associated with the glyph.
- **Molecular Species Glyphs** represent any class of molecule whose detailed structure is not being shown using sequence feature glyphs. They are associated with Systems Biology Ontology terms. For the SBOL 3 data model, this is formally defined as any `Feature` with a compatible term within its associated `types`, i.e., one that is equal to or a child of at least one term associated with the glyph.
- **Interaction Glyphs** are "arrows" indicating functional relationships between sequence features, molecular species, and/or other relationships. They are associated with Systems Biology Ontology terms. For the SBOL 3 data model, this is formally defined as any `Interaction` with a compatible term within its `types`, i.e., one that is equal to or a child of at least one term associated with the glyph, and with a compatible `Participation` at the head and tail of the arrow.
- **Interaction Node Glyphs** are placed at the junctions of edges to represent biochemical processes. They are associated with Systems Biology Ontology terms. For the SBOL 3 data model, this is formally defined as any `Interaction` with a compatible term within its `types`, i.e., one that is equal to or a child of at least one term associated with the glyph, and with a compatible `Participation` on the incoming and outgoing edges of the glyph

More than one glyph may share the same definition: in this case, these glyphs form a family of variants, of which precisely one MUST be designated as the RECOMMENDED glyph, which is to be used unless there are strong reasons to prefer an alternative variant.

It will also frequently be the case that a diagram element could be represented by more than one glyph (e.g., a glyph for a specific term and a glyph for a more general term). In such cases, it is RECOMMENDED that the most specific applicable glyph be used. However, if upward branching in the relevant ontology means two applicable glyphs do not have an ordered parent/child relation, then either MAY be used.

For example, a protein coding sequence (CDS) is a sequence feature that may be represented either using the CDS glyph (Sequence Ontology term SO:0000316) or the Unspecified glyph (Sequence Ontology term SO:0000001). Since SO:0000316 is contained by SO:0000001, the preferred glyph is CDS, rather than Unspecified. Likewise, a CDS may be represented by either a pentagonal glyph or an arrow glyph, but the pentagon is the RECOMMENDED variant, and so it is likewise preferred. Figure 1 illustrates this example.

Finally, note that the mapping from data model to glyph is not one-to-one: many SBOL 3 data model constructs can, at least in theory, be represented visually in multiple different ways. For example, a DNA construct carrying a heterologous gene could be represented by the molecular species glyph for a double-stranded nucleic acid, the sequence feature glyph for an engineered construct, or a series of sequence feature glyphs showing the internal structure of the gene. This ambiguity is deliberate allowing diagrams to select an appropriate level of detail for the information that a diagram is intended to convey.

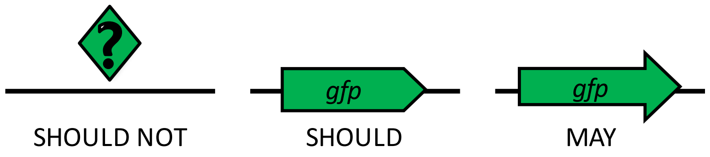

***Figure 1:*** *A biological design element such as a protein coding sequence (CDS) is best represented by the most specific RECOMMENDED glyph (middle), but can be represented by a less specific glyph such as Unspecified (left) or an approved alternative glyph (right).*

### 4.1 Requirements for Glyphs

A number of requirements are placed on all SBOL Visual glyphs in order to ensure both the clarity of diagrams and the ease with which they can be constructed:

1. A glyph SHOULD have its meaning defined by associating the glyph with at least one ontology definition. Definitions are RECOMMENDED to be from the Sequence Ontology for sequence feature glyphs, from the Systems Biology Ontology for molecular species glyphs, and from the Systems Biology Ontology for interaction glyphs. If no applicable terms are available in the preferred ontology, proposal of a new glyph SHOULD be accompanied by a request to the ontology maintainers to add a term for the undefined entity.
2. A glyph SHOULD be relatively easy to sketch by hand (e.g., no high-complexity images or precise angles required).
3. A glyph specification MUST indicate which portions of the glyph are the “interior” for purposes of color fill.
4. A glyph specification SHOULD show the glyph in its preferred relative scale with respect to other glyphs.
5. A glyph SHOULD be specified using only solid black lines (leaving color and style to be determined by the user, as noted below).
6. A glyph SHOULD NOT be similar enough to be easily confused with any other glyph when written by hand, or when scaled either vertically, horizontally, or both.
7. A glyph SHOULD NOT include text (note that associated labels are not part of the glyph).

In addition, some requirements apply only to certain classes of glyphs:

8. A sequence feature or molecular species glyph specification MUST include a rectangular bounding box indicating its extent in space.
9. A sequence feature glyph specification MUST include exactly one horizontal rule for its RECOMMENDED vertical alignment with the nucleic acid backbone.
10. A sequence feature glyph SHOULD be asymmetric on the horizontal axis. Vertical asymmetry is also preferred when possible.
11. If a sequence feature glyph can represent components of highly variable size or structural complexity, the glyph SHOULD be able to be scaled horizontally to indicate relative scale.

Figure 2 shows examples of compliant glyph specification.

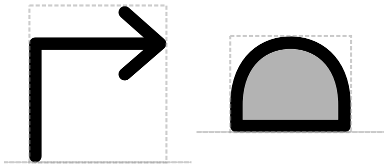

***Figure 2:*** *Examples of glyph specification: this specification for the sequence feature glyphs for Promoter (left) and Ribosome Entry Site (right) include the glyph outline, fill (grey center of Ribosome Entry Site), bounding box (dashed box), and recommended alignment with the nucleic acid backbone (dashed horizontal line), all at a preferred relative scale.*

### 4.2 Reserved Visual Properties

SBOL Visual aims to allow as much flexibility and freedom as possible in how diagrams are organized, presented, and styled. To this end, a number of aspects of presentation are generally reserved for the communication of other types of information by the creator of a diagram. When using a glyph in a diagram, the following choices in glyph presentation are thus explicitly intended to be alterable:

1. The lines of a glyph MAY be given any line thickness and style
2. The interior of a glyph MAY be given any fill color, as long as the choice of fill does not interfere with recognizing the glyph.
3. The scale of glyphs are RECOMMENDED to be kept consistent with their specification and throughout a diagram, but can be altered if desired, particularly to convey additional information (e.g., length of a sequence).
4. Minor styling effects MAY be chosen (e.g., shadow, corner styling, other "font-level" customization)

Figure 3 shows some examples of acceptable style variation.

In certain special cases, the style of a glyph may be more constrained, but such cases are expected to be rare and strongly motivated.

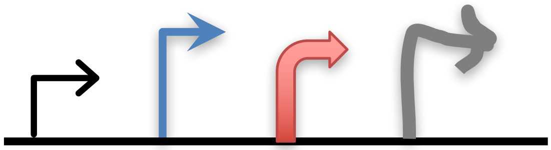

***Figure 3:*** *Examples of acceptable style variation for a Promoter glyph.*

### 4.3 Extending the Set of Glyphs

The collection of SBOL Visual glyphs is not expected to provide complete coverage of all of the types of element that people will wish to include in genetic diagrams, particularly given the ongoing evolution of synthetic biology as an engineering discipline. As the need for new diagram elements or new practices of usage emerge, new glyphs or glyph definitions are expected to be added to SBOL Visual. In particular, the following three classes of changes are expected to occur regularly, and the SBOL development community will maintain clear processes for proposal and adoption of changes of this type:

- New glyphs, either representing a type of component that previously lacked a glyph or enabling a distinction between types of components previously represented by the same glyph.

- Additional glyph variants, accompanied by compelling use cases that cannot be adequately addressed by the existing glyph variants.
- Additional definitions for a glyph, capturing an alternate meaning that is useful to humans but existing within a disjoint branch of the relevant ontology.

In order to support the coherent extension of SBOL Visual, whenever a diagram creator uses a glyph not found in Appendix A, the creator SHOULD submit it to be considered for inclusion in an updated version of the standard following the processes for adding new glyphs found on the community website at `http://sbolstandard.org`

## 5 SBOL Visual Diagram Language

An SBOL Visual diagram represents information about the structure of a biological design. SBOL Visual is particularly concerned with enabling clear communication about the structure of nucleic acid designs, though there is no requirement that a diagram include such.

If desired, an SBOL Visual diagram may also be associated with a machine-interpretable model (e.g., in SBOL, GenBank, or SBML format). In this document we describe the association with the SBOL 3 data mode (Baig et al., 2020)l, which provides a formal semantic grounding for all elements of an SBOL Visual diagram, but equivalent associations may be made between diagram elements and other models. In terms of the SBOL 3 data model, the description of a nucleic acid design is formally defined as a representation of a `Component`, the `Feature` objects describing its design, the `Interaction` and `Participation` objects describing their functional relationships, and the `Constraint` objects describing relationships of relative location, orientation, topology, and identity.

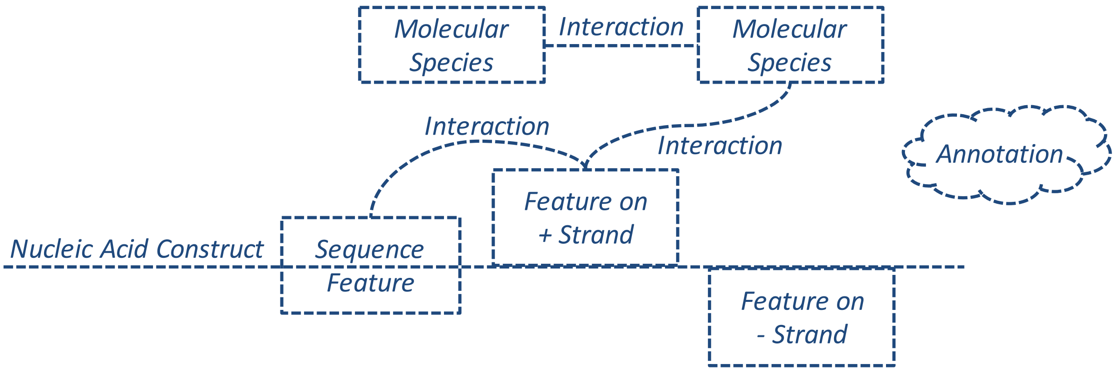

***Figure 4:*** *Generic syntax of SBOL Visual 3: a diagram for a nucleic acid construct is based around a backbone line, its structure specified by the sequence of attached sequence feature glyphs. Strand can optionally be indicated by placing a glyph above or below the backbone. Other molecular species are indicated by glyphs not in contact with any backbone. Interactions are directed edges connecting sequence feature or molecular species glyphs. Any of these objects may have an associated label showing its name, and the diagram may further include any form of other annotations, including other types of text.*

Specifically, an SBOL Visual diagram consists of the classes of objects illustrated in Figure 4. Figure 5 shows an example of such a diagram, in a typical usage. Full details of this specification are provided in the remainder of this section.

### 5.1 Nucleic Acid Backbone

A diagram for a nucleic acid construct is based around a single or double line, representing the nucleic acid backbone. Information about features of the construct can then be represented by attaching sequence feature glyphs to the backbone, as defined below in Section 5.2.

In terms of the SBOL 3 data model, the backbone represents any clustering of `Feature` objects that describe the structure of a single nucleic acid construct. In particular, a two `Feature` objects MAY be placed together on a nucleic acid backbone if:

- both `Feature` objects are children of the same `Component` with a nucleic acid `type` (e.g., DNA, RNA), or
- both `Feature` objects have a `Location` on the same nucleic acid `Sequence`, or
- the two `Feature` objects are linked together by a `Constraint` relation indicating their relative sequence location or orientation.

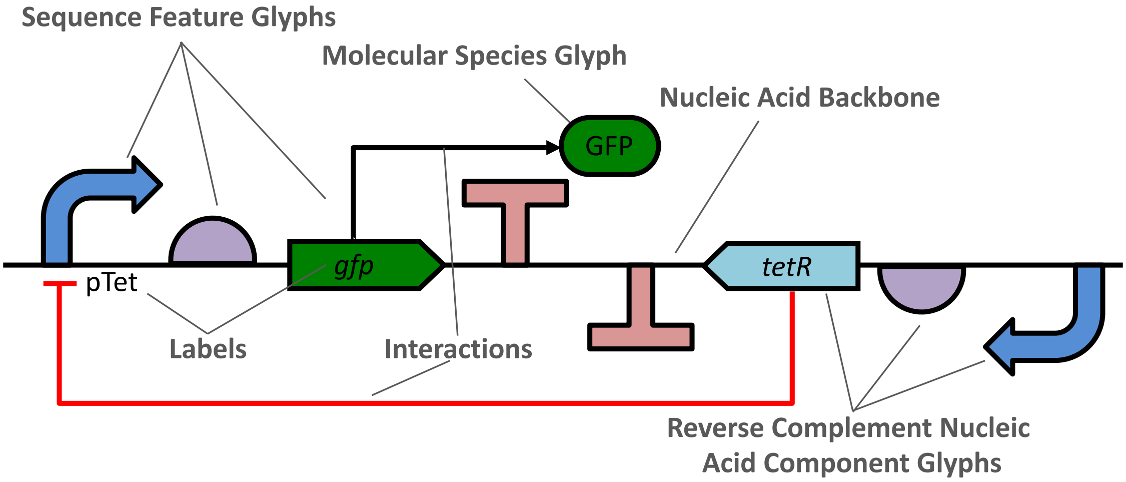

***Figure 5:*** *Example illustrating the elements of an SBOL Visual 2 diagram, with nucleic acid sequence features on the forward and reverse strand of a backbone, other molecular species, and interactions between elements; the grey labels and indicator lines are annotations.*

If two `Feature` objects do not have any such relation or chain of relations through other `Feature` objects, then they MUST NOT be placed on the same backbone.

The rules for use of nucleic acid backbones in diagrams are:

1. Lines in some cases indicate strand count. A double-stranded region of the nucleic acid construct MAY use either a single or double line for the backbone. A single-stranded region of the nucleic acid construct MUST use a single line to indicate the backbone. When single and double lines are mixed within a single diagram, the single lines always indicate single-stranded regions. Examples are provided in Figure 6.

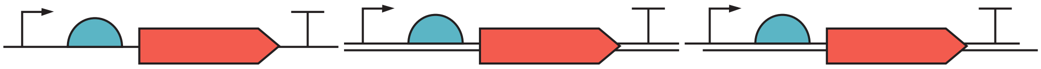

(a) Single- or double-strand backbone (b) Double-strand backbone (c) Double-strand backbone with single-strand overhangs

***Figure 6:*** *Examples of indicating strand count in nucleic acid backbones.*

2. A nucleic acid backbone SHOULD be horizontal in orientation, but MAY use non-horizontal structure to indicate important physical attributes (e.g., a closed loop to indicate a cyclic plasmid or more complex shapes for DNA nanotech structures). Examples are provided in Figure 7.

3. As a special case of non-horizontal backbone structure, certain stylized backbone shapes are used as sequence feature glyphs to indicate the genomic context of a sequence. These glyphs SHOULD be used as a matched pair, indicating the bounds of the context region. It is further RECOMMENDED that each glyph be concatenated with an Omitted Detail glyph to explicitly indicate that some surrounding context is not being shown. Examples are provided in Figure 8.

4. A nucleic acid backbone SHOULD have at least one associated feature glyph (else no structural information is being provided).

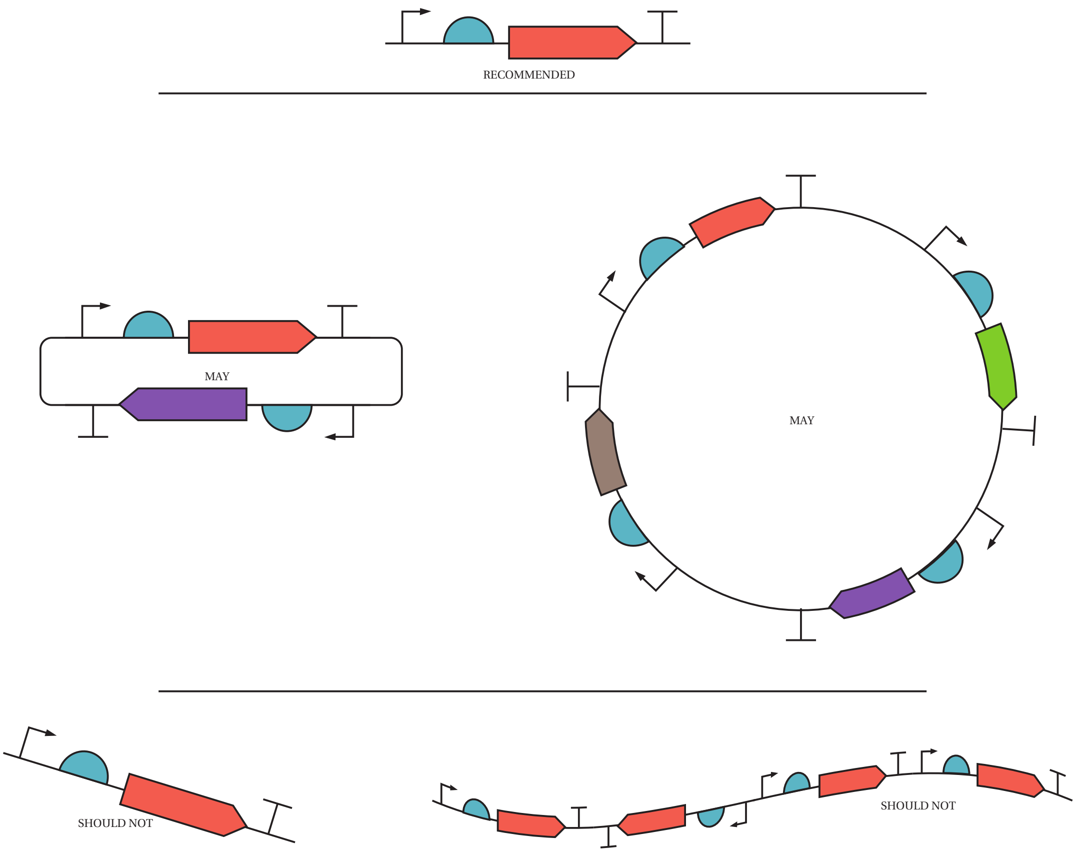

***Figure 7:*** *Recommended, acceptable, and problematic examples of nucleic backbone orientation.*

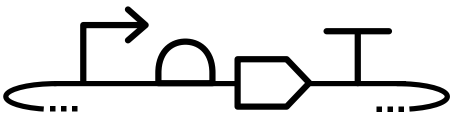

(a) Functional unit on a circular plasmid

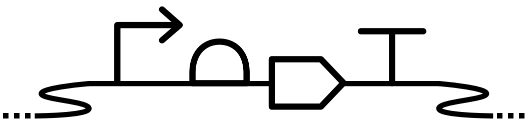

(b) Functional unit integrated at a chromosomal locus

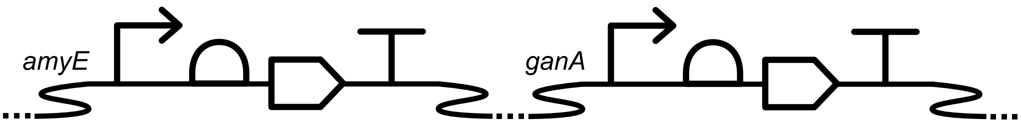

(c) Two functional units on the same chromosome but different loci.

***Figure 8:*** *Examples of RECOMMENDED indication of genomic context.*

### 5.2 Sequence Features

A sequence feature glyph in contact with a nucleic acid backbone indicates a feature of the nucleic acid sequence. In terms of the SBOL 3 data model, this is a `Feature` with a nucleic acid `type` that is associated with that nucleic acid backbone by the clustering rules given in Section 5.1. The `Feature` may be contained either directly, as one of the children of the `Component` represented by the diagram, or recursively through a sequence of `SubComponent` relationships.

1. Every feature glyph MUST have its bounding box in contact with the backbone for the nucleic acid construct it describes. The placement of the glyph SHOULD follow the recommendation for backbone alignment in the glyph specification. Examples are provided in Figure 9.

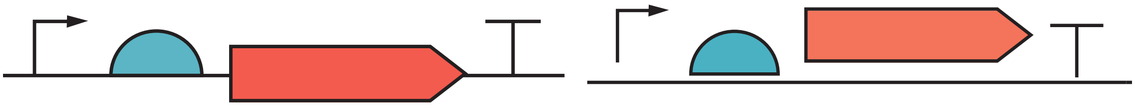

(a) MUST (b) MUST NOT

***Figure 9:*** *Examples of correct and incorrect association of glyphs with a nucleic acid backbone.*

2. The horizontal orientation of a glyph can be used to indicate the strand alignment of a feature, as shown in Figure 10. Any glyphs for a feature associated with the inline strand SHOULD be placed in the prototypical orientation given by the specification, while any glyph that is associated with the reverse complement strand SHOULD be inverted vertically and horizontally (i.e., rotated 180 degrees). Reverse complement MAY also be indicated by horizontal-only inversion. Finally, a glyph inverted only vertically still indicates inline strand, but it is RECOMMENDED NOT to use this orientation. Orientation SHOULD be used consistently throughout a diagram, rather than mixing conventions. Examples are provided in Figure 11.

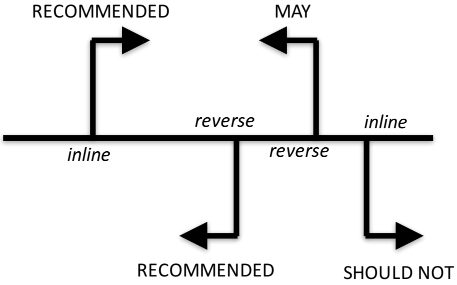

***Figure 10:*** *Use of glyph orientation to indicate inline vs. reverse complement direction.*

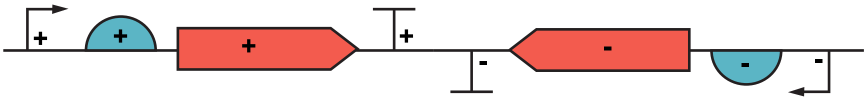

***Figure 11:*** *Example construct incorporating both inline (+) and reverse complement (-) features.*

3. Nucleic acid features in a sequential relationship SHOULD be drawn from 5' left to 3' right on the inline strand and from 5' right to 3' left on the reverse complement strand. In terms of the SBOL 3 data model, this indicates a `Constraint` on the relative ordering of two features.

4. Nucleic acid features that do not overlap in their locations SHOULD NOT have glyphs whose bounding boxes overlap. An example is provided in Figure 12.

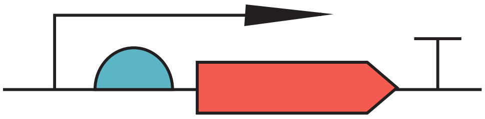

***Figure 12:*** *Example of incorrect glyph overlap: promoter (arrow) does not overlap in sequence with the ribosome entry site and CDS, so SHOULD NOT overlap visually with them.*

5. Nucleic acid features that overlap in their locations SHOULD have glyphs whose bounding boxes overlap. Overlap size MAY be used to indicate relative position. Examples are provided in Figure 13.

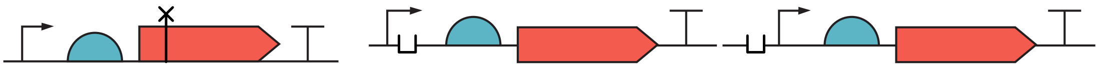

(a) Restriction site in a CDS (b) 3'-side operator in a promoter (c) 5'-side operator in a promoter

***Figure 13:*** *Examples where glyphs SHOULD overlap, but might not if it is more clear, e.g., with an operator site located within the 5' portion of a promoter.*

6. A nucleic acid feature SHOULD be represented using a glyph defined in Appendix A.1. In this case, the feature MUST be contained within at least one of the glyph's associated terms. In terms of the SBOL 2 data model, this means the glyph is equal to or a parent of at least one of the `roles` for the `Feature` or its associated `Component` if it is a `SubComponent`. Moreover, the glyph used SHOULD be the RECOMMENDED variant of the most specific applicable glyph. Note that novel glyphs not defined in Appendix A.1 MAY be used, but SHOULD be proposed for adoption as described in Section 4.3. Examples are provided in Figure 14.

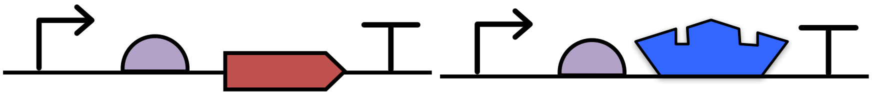

(a) SHOULD (b) MAY

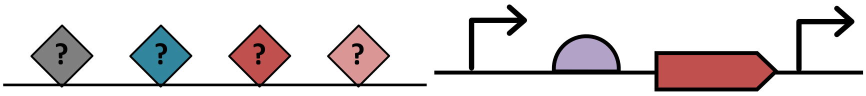

(c) SHOULD NOT (d) MUST NOT

***Figure 14:*** *Examples of recommended, allowed, and forbidden representation of a* `Component` *comprising a sequence of promoter, ribosome entry site, CDS, and terminator: (a) is RECOMMENDED because it uses the preferred variant of the most specific defined glyphs, (b) is allowed because it uses some novel custom non-conflicting symbol, not matching any glyph defined in this document, to encode more specific information about the particular CDS, (c) is recommended against because it uses less specific glyphs, and (d) is forbidden because it use a promoter symbol to represent the terminator.*

### 5.3 Molecular Species

A glyph that is not in contact with any backbone represents any class of molecule whose detailed structure is not being shown using sequence feature glyphs. In other words, either not a nucleic acid (e.g., proteins, small molecules) or else an “uninteresting” nucleic acid (e.g., showing a transcribed mRNA, but not the features of its sequence). In terms of the SBOL 3 data model, these are also `Feature` objects contained within the overall `Component` for the diagram.

1. A molecular species SHOULD be represented using a glyph defined in Appendix A.2. In this case, the species MUST be contained within at least one of the glyph’s associated SBO terms. In terms of the SBOL 3 data model, this means the SBO term for the glyph is equal to or a parent of at least one of the `types` for the associated `Feature` or its type-defining referent (i.e., the `Component` linked by `instanceOf` for a `SubComponent` or the type associated with the linked `Feature` for a `ComponentReference`). Moreover, the glyph used SHOULD be the RECOMMENDED variant of the most specific applicable glyph. Note that novel glyphs not defined in Appendix A.2 MAY be used, but SHOULD be proposed for adoption as described in Section 4.3.

2. The bounding box of a molecular species glyph MUST NOT contact any nucleic acid construct unless there is an interaction between the molecular species and the nucleic acid construct.

3. If a molecular species glyph overlaps a nucleic acid construct, then the location of the overlap SHOULD correspond with the location of an interaction on the nucleic acid construct. The molecular species glyph SHOULD be visually distinct from a sequence feature glyph. This location is RECOMMENDED to be represented with a sequence feature glyph appropriate to the interaction (e.g., a binding site or restriction site). Examples are provided in Figure 15.

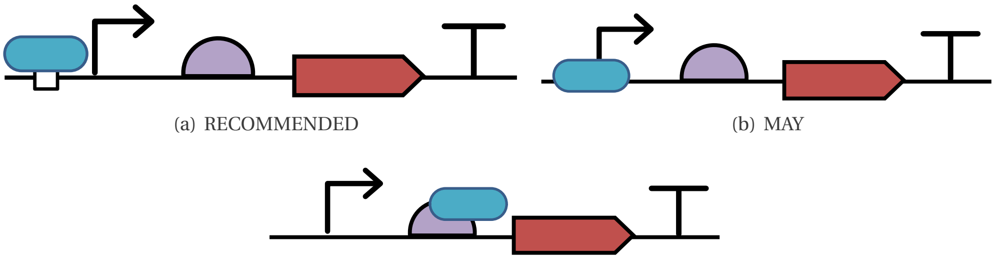

(a) RECOMMENDED

(b) MAY

(c) SHOULD NOT

***Figure 15:*** *Examples of recommended, allowed, and not recommended representation of an interaction between a molecular species and a nucleic acid construct, in this case regulation of a promoter by a transcription factor protein that binds on the 5’ side of the promoter: (a) shows the RECOMMENDED representation, (b) shows a more generic alternative, and (c) is recommended against because the location does not corresponding with the binding.*

### 5.4 Interactions

A directed edge “arrow” attached to one or more glyphs indicates a functional interaction involving those elements. The roles of the elements is indicated by their position at the head or tail of the edge. In terms of the SBOL 2 data model, this is an `Interaction`, with either one or two `Participation` relationships, their `role` set by position at the head or tail of the edge. An example is provided in Figure 16.

1. Two interaction edges SHOULD NOT cross one another. When edges cross, they MUST indicate the distinction between arrows with a crossover pattern, in which one edge “diverts” at the intersection (see Figure 17). Examples are provided in Figure 18.

2. An interaction SHOULD be represented using a glyph defined in Appendix A.3. In this case, the interaction type MUST be contained within at least one of the glyph’s associated terms. In terms of the SBOL 2 data

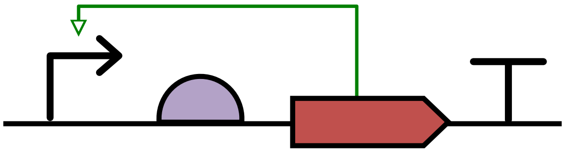

*Figure 16:* Example of an interaction indicating a promoter stimulated by the CDS that it regulates.

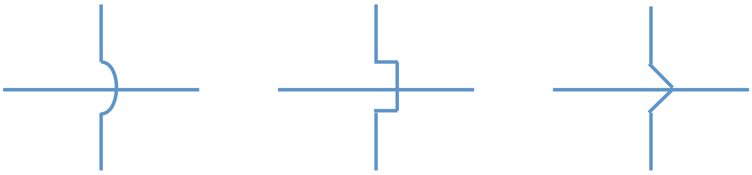

*Figure 17:* Examples of `Interaction` crossover patterns.

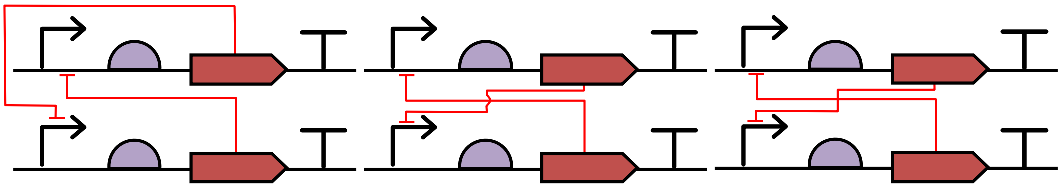

(a) SHOULD (b) MAY (c) MUST NOT

*Figure 18:* Examples of recommended, allowed, and forbidden relationships between two interactions in a mutual repression system: (a) non-crossing is recommended, (b) using a crossover pattern is allowed, but (c) crossing without a crossover pattern is forbidden, since the relationship between the two edges is ambiguous.

model, this means the glyph is equal to or a parent of at least one of the `types` for the `Interaction`, and that each associated `Participation` object has a `role` compatible with its position on the head or tail of the edge. Moreover, the glyph used SHOULD be the RECOMMENDED variant of the most specific applicable glyph. Note that novel glyphs not defined in Appendix A.3 MAY be used, but SHOULD be proposed for adoption as described in Section 4.3.

3. An edge may have multiple heads or multiple tails. In this case, a split or join in an edge represents either multiple participants with the same role (e.g., a transcription factor repressing two instances of a promoter) or a biochemical process (e.g., association of an inducible protein and a small molecule to form an active complex). An edge with multiple heads MUST use the same glyph for each head. An edge that splits or joins with no glyph at the junction represents multiple participants with the same role. Examples are provided in Figure 19.

4. A glyph at the point where an edge splits or joins represents a biochemical process, i.e., an `Interaction` with type and roles set by the process glyph. A biochemical process represented by a glyph at an edge junction SHOULD be represented using a glyph defined in Appendix A.4. In this case, the interaction type MUST be contained within at least one of the glyph's associated terms. In terms of the SBOL 2 data model, this means the glyph is equal to or a parent of at least one of the `types` for the `Interaction`, and that each associated `Participation` object has a `role` compatible with its position on the head or tail of the edge. Moreover, the glyph used SHOULD be the RECOMMENDED variant of the most specific applicable glyph. Note that novel glyphs not defined in Appendix A.4 MAY be used, but SHOULD be proposed for adoption as described in Section 4.3. Examples are provided in Figure 20.

5. An edge with its head at an interaction node MAY use an `Interaction` arrow head to indicate a `role` other than Reactant (SBO:0000010) in the biochemical process. Likewise, an edge with its tail at an interaction node MAY use an `Interaction` arrow head to indicate the `role` played by that product of the biochemical process in another `Interaction`. Examples are provided in Figure 21.

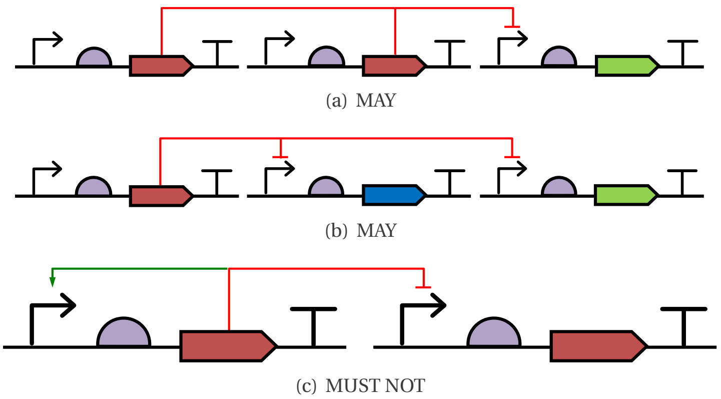

***Figure 19:*** *Examples of use of multi-head and multi-tail arrows: (a) Repression from multiple independent sources and (b) repressor with multiple targets. (c) Multi-head interactions, however, MUST NOT use different glyphs for different heads.*

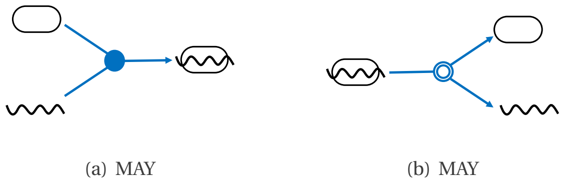

***Figure 20:*** *Examples of use of interaction nodes: (a) association of gRNA and Cas9 into an active CRISPR complex and (b) the dissociation of that complex.*

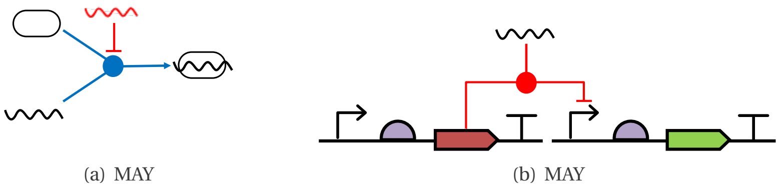

***Figure 21:*** *(a) Example of interaction node with an additional role indicated by an entering arrow head: association of gRNA and Cas9 inhibited by the presence of a competing gRNA. (b) Example of interaction node whose product plays a role in another interaction: dCas9 and gRNA associate to form a CRISPR complex, which then represses a promoter.*

6. An edge MAY have another edge at its head or tail, indicating that the interaction has a participant that is another interaction. This does not have a direct representation in the SBOL 2 data model. To avoid ambiguity, an edge SHOULD NOT connect to only one head of a multi-head arrow or one tail of a multi-tail arrow, and MUST connect only to the body of the edge, not its head or tail. Examples are provided in Figure 22.

### 5.5 Subsystems

A distinguished subsystem within a system MAY be represented by a visual boundary in the form of closed polygon or closed curve. Everything inside of the boundary is part of the subsystem, and everything outside of the boundary is not part of the subsystem; only certain diagram elements are allowed to cross a boundary, as defined below. In terms of the SBOL 3 data model, the line represents a `SubComponent` included within the `Component` represented by the surrounding diagram, and boundary-crossing elements define `ComponentReference` relationships. Note that the internals of a subsystem need not be shown: some details can be omitted or a subsystem can even be a “black box” with no internal structure at all being shown.

1. The boundary of subsystem SHOULD should be a rectangle or rounded rectangle. Boundary sides SHOULD be oriented vertically and horizontally. It is RECOMMENDED that a subsystem be made visually distinct by

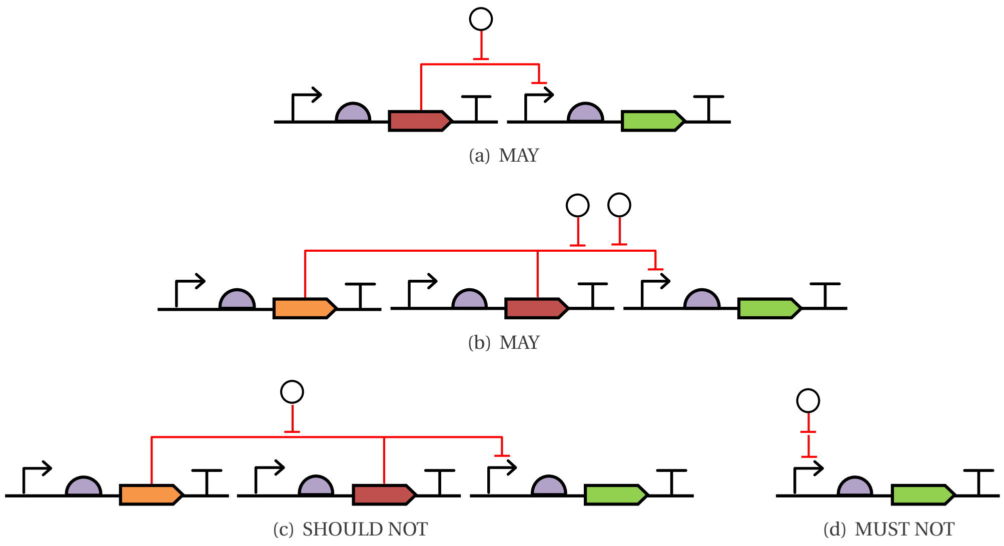

*Figure 22: Examples of allowed and forbidden use of interactions with interactions: (a) aTc inhibiting repression by TetR. (b) Multiple inhibitions of an interaction with two tails. (c) Interactions SHOULD NOT connect to only one head of a multi-head or tail of a multi-tail interaction. (d) interactions MUST NOT connect to the head or tail of another interaction.*

making it larger than other glyphs and with a different line style. Examples are provided in Figure 23.

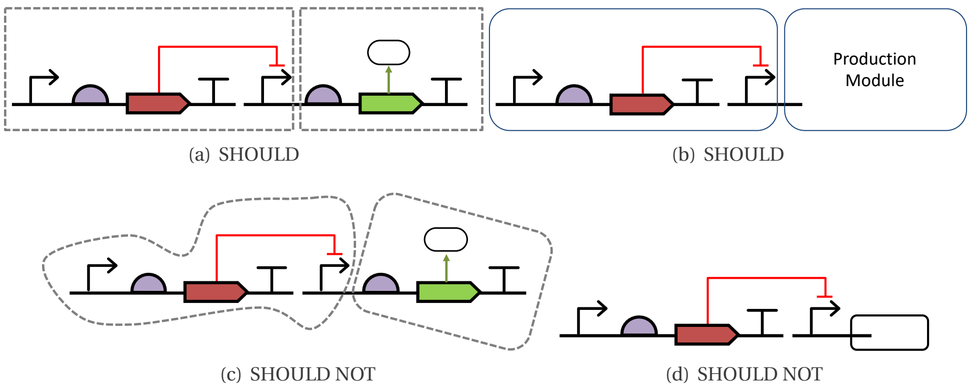

*Figure 23: Examples of recommended and problematic subsystem boundaries: (a) two subsystems with visually distinct rectangular borders, (b) shows the same subsystems but with rounded rectangles and the second being a "black box" subsystem with no internal structure shown, (c) shows subsystems with non-rectilinear borders, and (d) shows a black-box subsystem that is not visually distinct from a sequence feature glyph.*

2. Glyphs for sequence features and molecular species MUST NOT intersect with the boundary of a subsystem. An example is provided in Figure 24(a).

3. A nucleic acid backbone MAY cross the boundary of a subsystem. This represents an identification of the region of the nucleic acid construct contained within the subsystem and a compatible region of the larger construct represented in the enclosing system. In terms of the SBOL 3 data model, a nucleic acid backbone crossing a subsystem boundary represents the existence of `Constraint` or `Location` objects in the enclosing `Component` that indicate that the nucleic acid sequence in the `SubComponent` is adjacent to the connected portions of nucleic acid sequence in the larger `Component`. Examples are provided in Figure 24(b).

4. An interaction edge MAY cross the boundary of a subsystem. This represents an interaction in the enclosing system that references the connected feature in the subsystem. In terms of the SBOL 3 data model, an interaction edge crossing a subsystem boundary represents an `Interaction` in the enclosing `Component` with a `Participation` that is a `ComponentReference` to the indicated `Feature` in the `SubComponent`. Examples are provided in Figure 24(c).

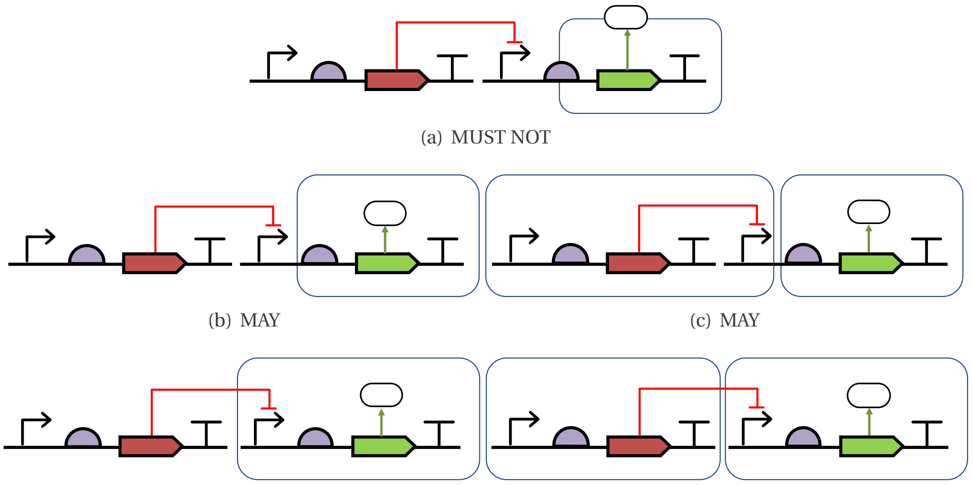

(a) MUST NOT

(b) MAY (c) MAY

(d) MAY (e) MAY

*__Figure 24:__ Examples of recommended and problematic boundary intersections: (a) sequence feature and molecular species glyphs MUST NOT intersect a subsystem boundary, (b) the repressed promoter and the regulated sequence elements in the subsystem are ordered to form a nucleic acid construct implementing a complete functional unit, (c) the promoter in the left subsystem and the regulated elements in the right subsystem are ordered to form a nucleic acid construct implementing a complete functional unit, (d) Interaction in which the CDS inhibits the promoter in the subsystem (presumably via a repressor product), (e) Interaction in which the CDS in the left subsystem inhibits the promoter in the right subsystem (presumably via a repressor product).*

5. Small rectangles MAY be drawn on the outside of the subsystem boundary to represent its intended interface. In terms of the SBOL 3 data model, these represent the `Interface` for the `SubComponent`, with each rectangle associated with a `Feature` designated as an `input`, `output`, or `nondirectional` element of the interface. An interface rectangle may be connected to an interaction edge head or tail to represent interactions with its associated feature. If both an interface rectangle and a glyph for its associated feature are present in a diagram, then any interaction with the feature from outside of the subsystem MUST both pass through the interface rectangle and connect with the glyph. An interaction with a feature that does not pass through an interface rectangle then MAY be used to represent an unintended interaction with a non-interface element of the subsystem. Examples are provided in Figure 25.

### 5.6 Labels

The name of any object in a diagram is RECOMMENDED to be displayed as text within, adjacent to, or otherwise clearly visually connected to the object's associated glyph. In terms of the SBOL 3 data model, this is the `name` property, and if no `name` is supplied then the `displayId` MAY be used instead. Examples are provided in Figure 26.

### 5.7 Annotations

Other text or graphics may be included as annotations with no constraint on their syntax or semantics.

1. Annotations SHOULD NOT be displayed in a way that allows them to be confused with other SBOL Visual elements.

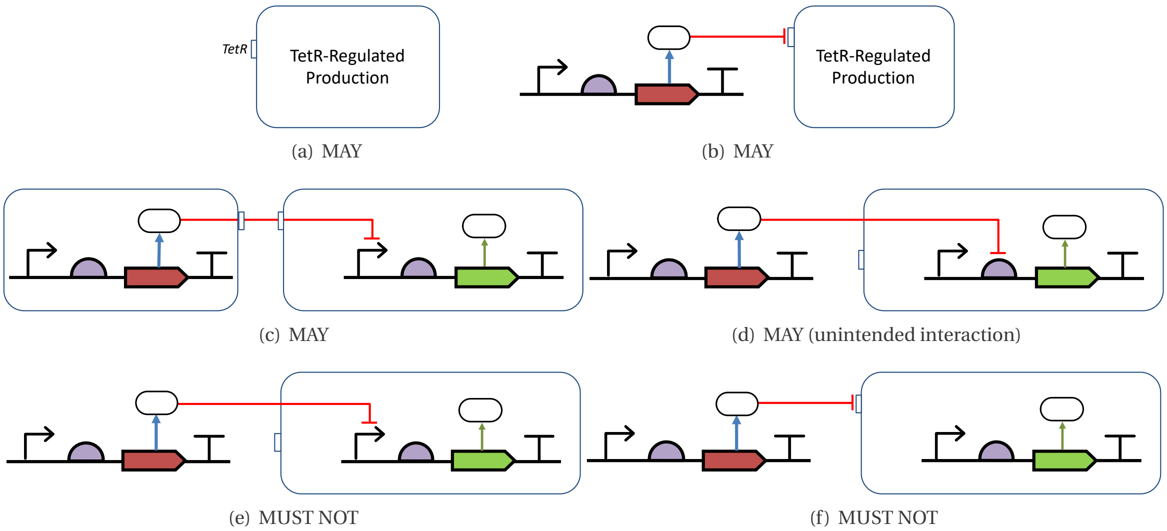

***Figure 25:*** *Examples of recommended and problematic subsystem interfaces: (a) interface on a black-box subsystem, (b) interaction with a black-box interface, (c) boundary-crossing interaction passing through an interface, (d) unintended interaction with a non-interface element, but (e) interactions with features in the interface must pass through the interface rectangle and (f) when the subsystem is shown, interactions MUST NOT terminate at the interface*

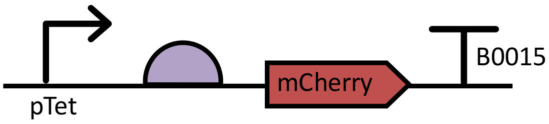

***Figure 26:*** *Examples of labels on glyphs.*

2. Annotations SHOULD NOT be used to display information that can be displayed using other SBOL Visual elements.

### 5.8 Criteria for Compliance with SBOL Visual

A diagram of a biological system is compliant with SBOL Visual if it complies with all MUST and MUST NOT requirements as specified above. A diagram is compliant with SBOL Visual best practices if it also complies with all RECOMMENDED, SHOULD, and SHOULD NOT statements as specified above.

Importantly, note that a non-SBOL glyph can be used in a compliant diagram when its definition is a subset or superset of a definition that does have an SBOL Visual glyph. For example, a diagram that creates a new glyph for a special type of promoter can be SBOL Visual compliant even though there is an SBOL Visual glyph for a general promoter.

A piece of software or other system for producing diagrams is compliant with SBOL Visual under the following conditions:

1. The system MUST be capable of producing diagrams that are compliant with SBOL Visual.
2. If the system can also produce diagrams that are *not* compliant with SBOL Visual, it MUST clearly distinguish to the user between compliant and non-compliant usage and diagrams.

## A SBOL Visual Glyphs

The following pages present all current glyphs for SBOL Visual, organized by glyph families. Each entry lists:

- Glyph family name
- Associated ontology terms
- Recommended and alternate glyphs
- At least one example of when this glyph would be used
- Any additional notes

### A.1 Sequence Feature Glyphs

These glyphs represent features of nucleic acid sequences, and include a bounding box (grey dashed box) and a recommended alignment to the nucleic acid backbone (grey dashed horizontal line).

## Aptamer 1

### Associated SO term(s)

SO:0000031: Aptamer

### Recommended Glyph and Alternates

The aptamer glyph is a cartoon diagram of a prototypical nucleic acid secondary structure for an aptamer:

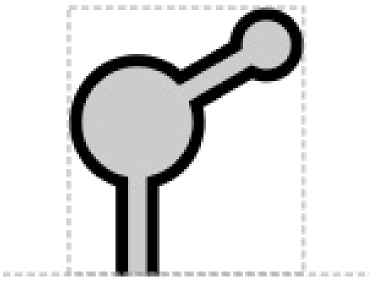

### Prototypical Example theophylline aptamer

### Notes

*this section deliberately blank*

## Assembly Scar

1

### Associated SO term(s)

SO:0001953

### Recommended Glyph and Alternates

The assembly scar glyph is an "equal sign" image, the pattern produced by the union of a 5' sticky end and 3' sticky end glyph. The scar will cover the backbone, creating a visual break suggesting the potential disruption associated with a scar:

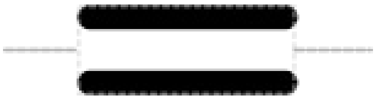

With a double-stranded backbone:

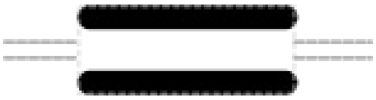

### Prototypical Example

Ligated sticky ends following BioBrick assembly.

### Notes

*this section deliberately blank*

## Blunt Restriction Site 1

### Associated SO term(s)

SO:0001691

### Recommended Glyph and Alternates

The blunt restriction site glyph is an image of two brackets facing away from one another to make a smooth-edged gap:

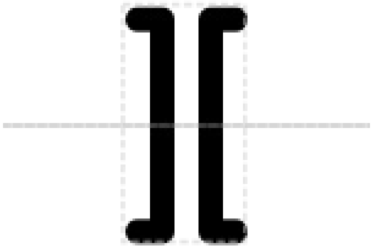

### Prototypical Example

EcoRV restriction site

### Notes

*this section deliberately blank*

## CDS 1

### Associated SO term(s)

SO:0000316

### Recommended Glyph and Alternates

The coding sequence glyph is a "box" with one side bent out arrow-like to show direction:

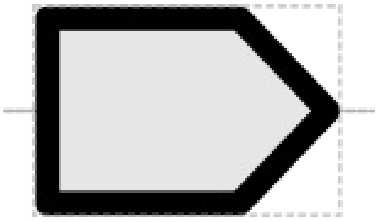

Alternately, CDS may be represented as a block arrow:

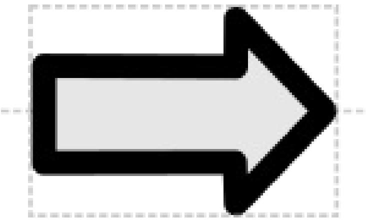

### Prototypical Example

α-Hemoglobin coding sequence

### Notes

*this section deliberately blank*

## Chromosomal Locus

### Associated SO term(s)

SO:0000830 Chromosome Part

### Recommended Glyph and Alternates

The glyph to indicate integration into a chromosome is an S-shaped curve of the backbone, suggesting something that might be part of a larger looping structure:

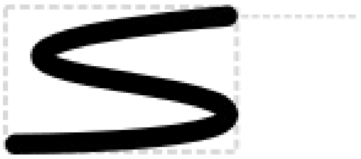

### Prototypical Example

*B. subtilis* amyE locus

### Notes

Complementary "left" and "right" versions of this glyph SHOULD be used together, flanking the region whose genomic context is being described.

The Omitted Detail glyph SHOULD generally be contatenated to indicate that there is information about the chromosome not being represented.

Examples of RECOMMENDED usage:

- A functional unit consisting of promoter, ribosome entry site, CDS, and terminator, all integrated together into the chromosome:

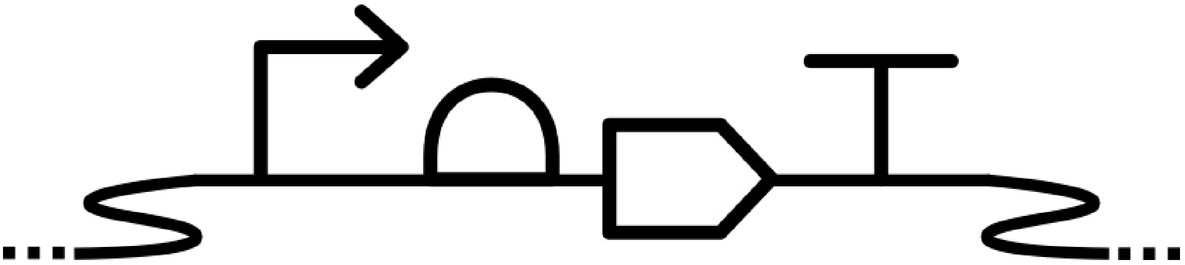

- Two functional units, one integrated into the amyE locus, another integrated into the ganA locus:

## Circular Plasmid 1

### Associated SO term(s)

SO:0002211 Circular Plasmid - A self replicating circular nucleic acid molecule that is distinct from a chromosome in the organism.

### Recommended Glyph and Alternates

The glyph to indicate embedding in a plasmid is a turn of the backbone indicating its circular structure:

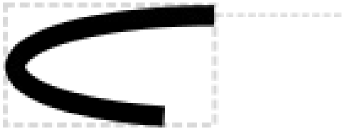

### Prototypical Example

*E. coli* p15A plasmid

### Notes

Note that for SBOL data representations, circularity SHOULD also be indicated with a type of SO:0000988.

Complementary "left" and "right" versions of this glyph SHOULD be used together, flanking the region whose genomic context is being described.

The Omitted Detail glyph SHOULD generally be concatenated to indicate that there is information about the plasmid not being represented.

Example of RECOMMENDED usage: a plasmid containing a functional unit consisting of promoter, ribosome entry site, CDS, and terminator:

## Cleavage Site

### Associated SO term(s)

SO:0001688 (Restriction Enzyme Cleavage Junction), SO:0001687 (Restriction Enzyme Recognition Site)

SO:0001977 (Ribonuclease Site)

SO:0001956 (Protease Site)

### Recommended Glyph and Alternates

Cleavage Site is a "stem-top" glyph for describing small sites. In this system:

- the top glyph indicates the type of site (e.g., Cleavage Site)
- the stem glyph indicates whether the site affects DNA, RNA, or protein (respectively: straight, wavy, or looped)

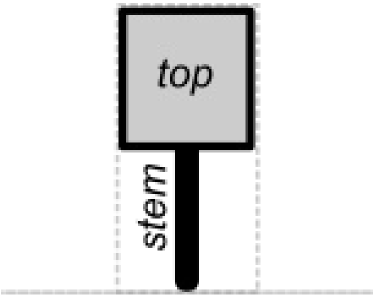

The Cleavage Site top is an "X" suggesting slicing on top of a stem connecting to the backbone at the point where cleavage will occur (in order: DNA, RNA, Protein):

### Prototypical Example

RNAse E site, BamHI

### Notes

SO:0000061 (which was previously associated with Restriction Enzyme Recognition Site in SBOL Visual 1) is no longer associated with the DNA Cleavage glyph in SBOL Visual 2 or 3, as SO:0000061 refers to the binding site and not the location of cleavage.

The Ribonuclease Site, Protease Site, and Restriction Enzyme Recognition Site glyphs from SBOL Visual 1.0 are now replaced by the Cleavage Site glyph with the appropriate stem.

Describing a Restriction Enzyme Cleavage Site with a vertical line glyph on a DNA backbone (as done previously in SBOL Visual 1.0 via the Restriction Enzyme Recognition Site glyph) can persist in a SBOL Visual 2 or 3 diagram and still be considered compliant with SBOL Visual 2 or 3, where it is now classified as a Biopolymer Location (which is a superclass of cleavage sites). Thus, the Biopolymer Location glyph from SBOL Visual 2 or 3 is backwards compatible with the Restriction Enzyme Recognition Site glyph from SBOL Visual 1.

The 5' Sticky Restriction Site, 3' Sticky Restriction Site, and Blunt Restriction Site glyphs remain unchanged, and are more specific children/derivatives of the DNA-Stem Cleavage-Top glyph.

A 2A self-cleaving polypeptide region (SO:0002224) SHOULD NOT be represented by a protease site, as its cleavage mechanism is different. Instead, 2A sequences should be represented using the Polypeptide Region glyph (see example in its specification). 1

## Composite

### Associated SO term(s)

*Composite does not have an associated SO term, as it merely links a base glyph (with its own SO term) to a sub-diagram (comprising glyphs with their own associated SO terms).*

### Recommended Glyph and Alternates

The glyph for Composite is dashed "expanding lines" connecting any "base" glyph representing the more abstract composite (e.g., Omitted Detail, or Terminator, or Promoter) to a backbone diagramming the contents of the composite. Note the bounding box is indicating the location of the base glyph, and would scale with that glyph.

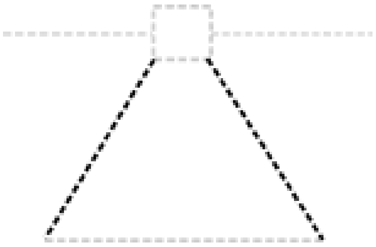

### Prototypical Example

An "expression cassette" containing a ribosome entry site, coding sequence, and terminator.

In this case, the recommended "base" glyph would be Engineered Region.

### Notes

An "abbreviated" representation of composite, simply indicating that more structure is available, can be made by using short lines and placing only an Omitted Detail glyph in the secondary backbone. For example, here is an example of an abbreviated composite promoter:

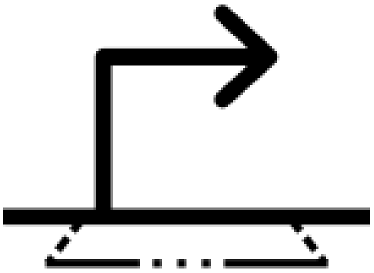

and a composite with an Engineered Region of otherwise unspecified content:

1

## Engineered Region

1

### Associated SO term(s)

SO:0000804 (Engineered Region)

### Recommended Glyph and Alternates

Engineered Region is represented by a plain rectangle suggesting a blank slate to be written upon:

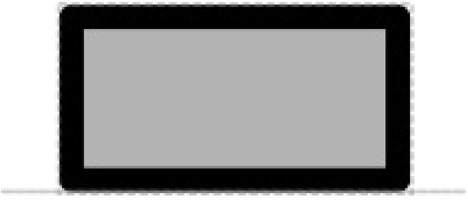

### Prototypical Example

An "expression cassette" containing a ribosome entry site, coding sequence, and terminator.

### Notes

*this section deliberately blank*

## Inert DNA Spacer

1

### Associated SO term(s)

SO:0002223 (Inert DNA Spacer)

### Recommended Glyph and Alternates

The inert DNA spacer glyph is a circle with an X in its middle, suggesting the intent to cancel possible interactions:

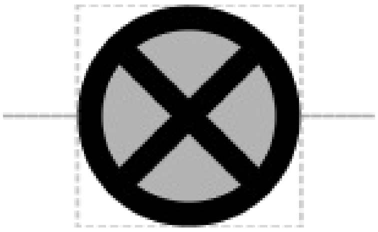

### Prototypical Example

Inserted 5' sequence intended to reduce effect of upstream genetic context on promoter behavior.

### Notes

*this section deliberately blank*

## Intron

1

### Associated SO term(s)

SO:0000188 (intron)

### Recommended Glyph and Alternates

An intron is designated by a boundaries interrupting CDS, each side having a two-triangle "torn out" edges, suggesting removal from an enclosing coding sequence:

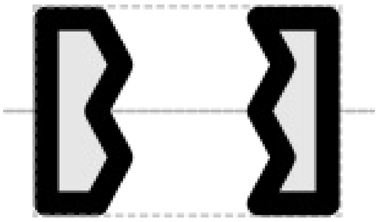

### Prototypical Example

Example of a coding sequence with three domains: an N-tag (blue), C-tag (yellow), and internal region (red) interrupted by an intron that includes a gRNA non-coding RNA sequence (green):

### Notes

*this section deliberately blank*

## Biopolymer Location

### Associated SO term(s)

DNA: SO:0000699 (Junction, Boundary, Breakpoint), SO:0001236 (Base)

RNA: SO:0000699 (Junction, Boundary, Breakpoint), SO:0001236 (Base)

Protein: SO:0000699 (Junction, Boundary, Breakpoint), SO:0001237 (Amino Acid)

### Recommended Glyph and Alternates

Biopolymer Location is a "stem-top" glyph for describing small sites. In this system:

- the top glyph indicates the type of site (e.g., Biopolymer Location)
- the stem glyph indicates whether the site affects DNA, RNA, or protein (respectively: straight, wavy, or looped)

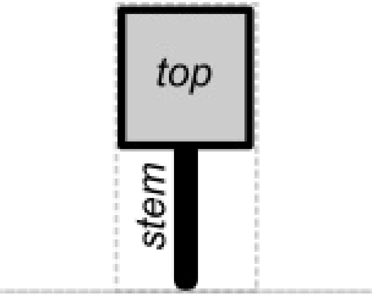

The RECOMMENDED top for Biopolymer Location is a circle, reminiscent of a pin stuck into a location (in order: DNA, RNA, Protein):

1

An alternative is to have "nothing" for the top, just an extended version of the stem itself (in order: DNA, RNA, Protein):

### Prototypical Example

1

CRISPR-targeted insertion site, protease site, mutation site

### Notes

Biopolymer Location is a general glyph for all zero- and one-length sequence features, including insertion and deletion sites and X-ase cut sites.

Note also that Biopolymer Location does not cover stability elements, since their length is typically multiple bases / amino acids.

Describing a Restriction Enzyme Cleavage Site with a vertical line glyph on a DNA backbone (as done previously in SBOL Visual 1 via the Restriction Enzyme Recognition Site glyph) can persist in a SBOL Visual 2 or 3 diagram and still be considered compliant with SBOL Visual 2 or 3, where it is now classified as a Biopolymer Location (which is a superclass of cleavage sites). Thus, the Biopolymer Location glyph from SBOL Visual 2 or 3 is backwards compatible with the Restriction Enzyme Recognition Site glyph from SBOL Visual 1.

## No Glyph Assigned

### Associated SO term(s)

Any SO term that is not covered by any glyph besides the root Sequence Feature

### Recommended Glyph and Alternates

When a part has no assigned glyph it is RECOMMENDED that a user provide their own glyph. The user is also encouraged to submit the new glyph for possible adoption into the SBOLv standard.

An alternative is brackets, suggesting information that needs to be filled in:

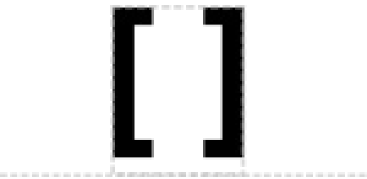

As a best practice, it is RECOMMENDED that the name of the term be put in between the brackets.

### Prototypical Example

No Glyph Assigned is intended to be used for any Component that is not covered by other SBOL Visual glyphs.

For example, at present there is no glyph recommended for representing a transposon.

### Notes

No Glyph Assigned is intended for constructs with a defined specific role that happens to not yet be covered by available approved glyphs (other than the root "Sequence Feature"). It is more likely to appear in machine-generated diagrams than in human-generated diagrams, since humans are likely to invent and use their own glyph for the purpose.

## Non-Coding RNA Gene

1

### Associated SO term(s)

SO:0001263: Non-Coding RNA Gene

SO:0000834: Mature Transcript Region

### Recommended Glyph and Alternates

The non-coding RNA glyph is a rectangular box whose top is a single-stranded RNA "wiggle":

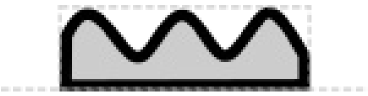

### Prototypical Example gRNA sequence for targeting a dCas9 repressor

### Notes

*This section left deliberately blank*

## Omitted Detail

### Associated SO term(s)

No SO term is associated with Omitted Detail, as it is indicating that something is *not* being represented.

### Recommended Glyph and Alternates

The Omitted Detail glyph is a break in the backbone with an ellipsis to indicate that material would normally be in that location:

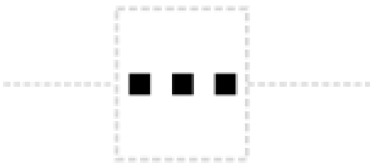

### Prototypical Example

A diagram in which a sequence feature is not drawn.

### Notes

This glyph actually places a "break" in the nucleic acid backbone.

## Operator / Binding Site

### Associated SO term(s)

SO:0000057 Operator

SO:0000409 Binding Site

### Recommended Glyph and Alternates

The operator glyph is an open "cup" suggesting a binding location:

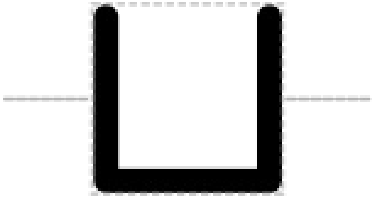

### Prototypical Example

Gal4 binding site in an activatable promoter.

### Notes

This glyph puts a "dent" in the backbone line.

## Origin of Replication

1

### Associated SO term(s)

SO:0000296

### Recommended Glyph and Alternates

The origin of replication glyph is a circle suggesting the "bulge" opened in a piece of circular DNA when replication is beginning:

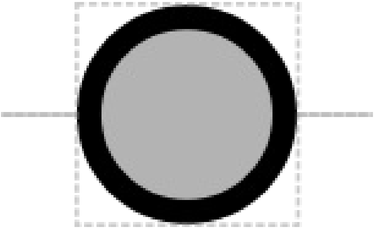

### Prototypical Example human herpesvirus-6 OOR

### Notes

The label on an origin of replication glyph is RECOMMENDED as the location to label either specifically the identity of the origin of replication or the name of the entire plasmid backbone more generally.

## Origin of Transfer

1

### Associated SO term(s)

SO:0000724: Origin of Transfer

### Recommended Glyph and Alternates

The origin of transfer glyph is circular like origin of replication, but also includes an outbound arrow:

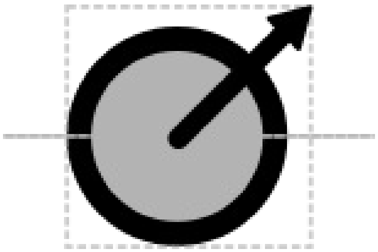

### Prototypical Example oriT

### Notes

*This section left deliberately blank*

## Overhang Site

### Associated SO term(s)

SO:0001932: 5' Overhang Site

SO:0001933: 3' Overhang Site

### Recommended Glyph and Alternates

The 5' overhang site glyph is an image of a strand of DNA extended on the 5' edge of its forward strand, and the complementary 3' Overhang Site glyph is a reflection of the 5' Overhang Site (in order: five-prime, three-prime):

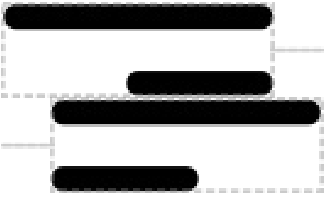

With a double-stranded backbone (in order: five-prime double stranded, three-prime double stranded):

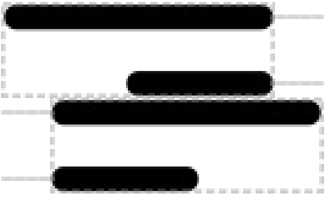

### Prototypical Example

EcoRI site after cleavage.

## PolyA Site

1

### Associated SO term(s)

SO:0000553: polyA Site

### Recommended Glyph and Alternates

The polyA site glyph is a sequence of As sitting atop the backbone:

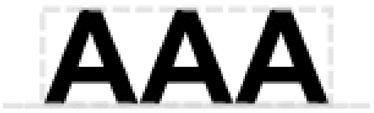

### Prototypical Example polyA tail on mammalian coding sequence

### Notes

*This section left deliberately blank*

## Polypeptide Region 1

### Associated SO term(s)

SO:0000839 (polypeptide region)

### Recommended Glyph and Alternates

A polypeptide region inside a coding sequence is indicated by insertion of triangular boundaries inside of the CDS, parallel to the 3' side of the CDS. This will produce chevron segments on the 3' side and a CDS shape on the 5' side:

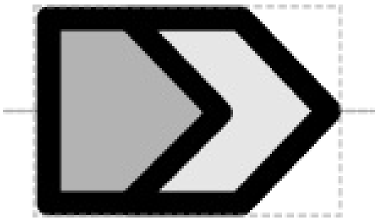

### Prototypical Example degradation tag on a protein coding sequence nuclear localization tag on a protein coding sequence coding sequence for the membrane-crossing region of a protein

This glyph is intended to be used in composition or superposition with the glyph for the coding sequence of which the polypeptide regions are fragments: Example of a coding sequence with three designated domains, an N-tag (blue), C-tag (yellow), and internal region (red):

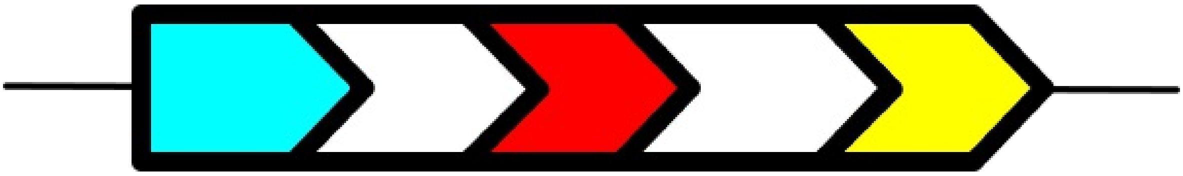

### Notes

Polypeptide region can also be used to represent regions that involve cleavage, such as a 2A self-cleaving polypeptide region (SO:0002224, a child term of SO:0000839). It is RECOMMENDED that cleavage-inducing polypeptide regions be visually distinguished from intact, e.g., through the use of dashed lines.

1

## Primer Binding Site

### Associated SO term(s)

SO:0005850

### Recommended Glyph and Alternates

The primer binding site glyph is a line with a bent end suggesting a partially complementary strand of nucleic acid attaching to the backbone:

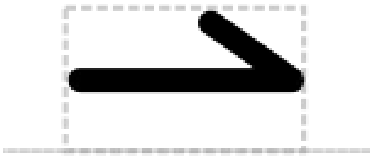

### Prototypical Example seq-F

### Notes

*this section deliberately blank*

## Promoter Site

### Associated SO term(s)

SO:0000167

### Recommended Glyph and Alternates

The promoter glyph is a bent arrow pointing forward, suggesting the action of transcription from its transcription start site:

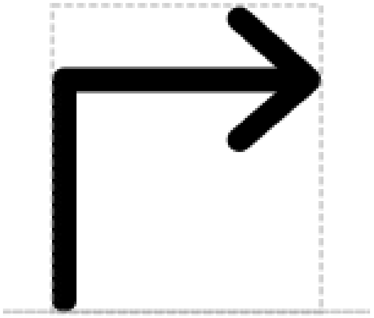

### Prototypical Example

The lacYZA promoter

### Notes

*this section deliberately blank*

## Ribosome Entry Site

1

### Associated SO term(s)

SO:0000139: Ribosome Entry Site

### Recommended Glyph and Alternates

The ribosome entry promoter glyph is a half-ovoid sitting on the backbone, suggesting an attached ribosome beginning transcription:

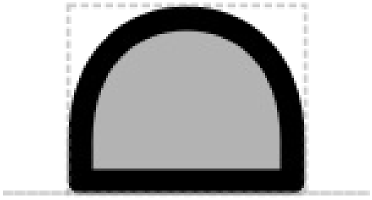

### Prototypical Example

T7g10 ribosome binding site

### Notes

*this section deliberately blank*

## Signature

### Associated SO term(s)

SO:0001978

### Recommended Glyph and Alternates

The signature glyph is a box sitting atop the backbone with an X and line inside it, suggesting a signature on a form:

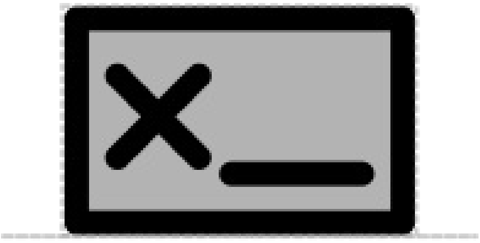

### Prototypical Example

DNA Barcode

### Notes

*this section deliberately blank*

## Specific Recombination Site 1

### Associated SO term(s)

SO:0000299: Specific Recombination Site

### Recommended Glyph and Alternates

The specific recombination site glyph is a triangle, centered on the backbone, as has appeared in a number of recombinase circuit papers:

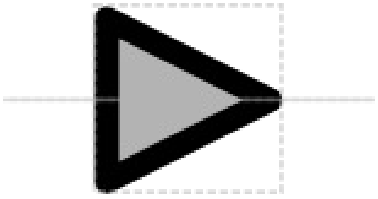

### Prototypical Example flippase recognition target (FRT) site

### Notes

*This section left deliberately blank*

## Stability Element 1

### Associated SO term(s)

*No SO term is currently associated with DNA stability.*

SO:0001979 (RNA Stability Element)

SO:0001955, SO:0001546 (Protein Stability Element)

### Recommended Glyph and Alternates

Stability Element is a "stem-top" glyph for describing small sites. In this system:

- the top glyph indicates the type of site (e.g., Stability Element)
- the stem glyph indicates whether the site affects DNA, RNA, or protein (respectively: straight, wavy, or looped)

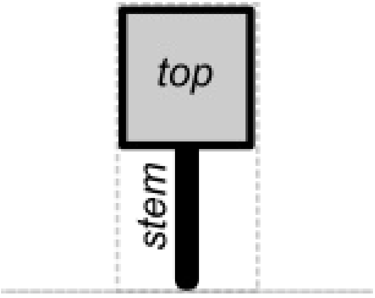

The top for a Stability Element is a pentagon suggesting the shape of a shield, on top of a stem connecting to the backbone at the point where the stability element is located (in order: DNA, RNA, Protein):

1

### Prototypical Example

PEST tag, 3’ Hairpin

### Notes

RNA Stability Element glyph was previously also associated with SO:0001957, but that SO term has been declared obsolete in Sequence Ontology.

This glyph is not backwards compatible with SBOL Visual 1.

Despite both being stem-top glyphs, Biopolymer Location is not a parent to Stability Element, since the length of a Stability Element is typically multiple bases / amino acids.

## Sticky End Restriction Enzyme Cleavage Site

1

### Associated SO term(s)

SO:0001975 (5' Sticky Restriction Site)

SO:0001976 (3' Sticky Restriction Site)

### Recommended Glyph and Alternates

The 5' sticky restriction site glyph is an image of the lines along which two strands of DNA will be cut into 5' sticky ends, and the complementary 3' Sticky Restriction Site glyph is a reflection of the 5' Sticky Restriction Site. Vertical position with respect to the backbone is in a break in a single backbone (in order: five-prime, three-prime):

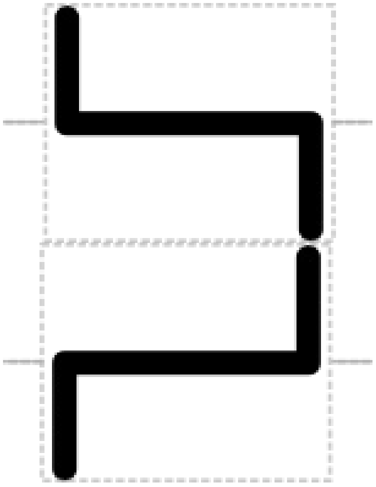

and between strands of a double backbone (in order: five-prime double stranded, three-prime double stranded):

### Prototypical Example

EcoRI restriction site.

### Notes

*this section deliberately blank*

## Stop Site 1

### Associated SO term(s)

SO:0000616 Transcription End Site

SO:0000319 Stop Codon, SO:0000327 Coding End, Translation Termination Site, Translation End

### Recommended Glyph and Alternates

Transcription/Translation End Point is a "stem-top" glyph for describing small sites. In this system:

- the top glyph indicates the type of site (e.g., Biopolymer Location)
- the stem glyph indicates whether the site affects DNA, RNA, or protein (respectively: straight, wavy, or looped)

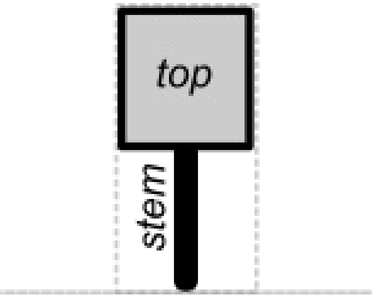

The Transcription/Translation End Point top is an asterisk in a circle (in order: transcription, translation):

### Prototypical Example

Location where a terminator causes transcription to stop, stop codon

### Notes 1

Note that the number of points in the asterisk is not specified, accomodating font differences.

## Terminator 1

### Associated SO term(s)

SO:0000141: Terminator

### Recommended Glyph and Alternates

The terminator is a T sitting atop the backbone:

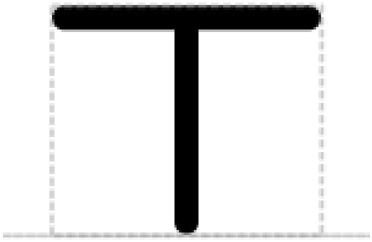

### Prototypical Example

T1 terminator

### Notes

*this section deliberately blank*

## Unspecified

1

### Associated SO term(s)

Unspecified: SO:0000110 Sequence Feature

### Recommended Glyph and Alternates

Unspecified is RECOMMENDED to be represented by the unicode "replacement character" glyph, indicating a missing or invalid symbol:

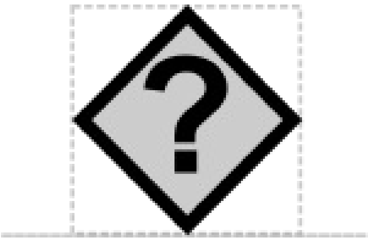

A half-rounded rectangle, the SBGN glyph for a nucleic acid, is an alternative:

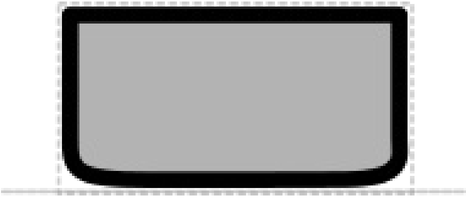

### Prototypical Example

An anonymous sequence that is missing any information about its nature or intended purpose.

### Notes

The Unspecified glyph is intended for showing where a sequence's role is missing (or, equivalently, given only the uninformative "Sequence Feature" root role). It should never appear with well-curated designs or diagrams.

### A.2 Molecular Species Glyphs

These glyphs represent molecular species in a diagram, and include a bounding box (grey dashed box) but are not connected to any nucleic acid backbone.

## Complex

### Associated SBO term(s)

SBO:0000253 Non-covalent complex

### Recommended Glyph and Alternates

The RECOMMENDED glyph for a complex is a composite of the glyphs for the molecules comprising the complex. For example, a protein bound to a simple chemical, a guide RNA, or another protein (in order:protein-small molecule, protein-guide RNA, protein-protein):

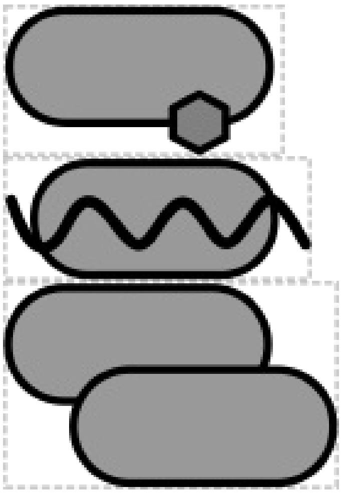

This may also be applied to show complex formation (binding) of a molecule to a nucleic acid construct by compositing the molecule glyph with the appropriate portion of the nucleic acid construct. For example, a protein binding to the promoter of a transcriptional unit:

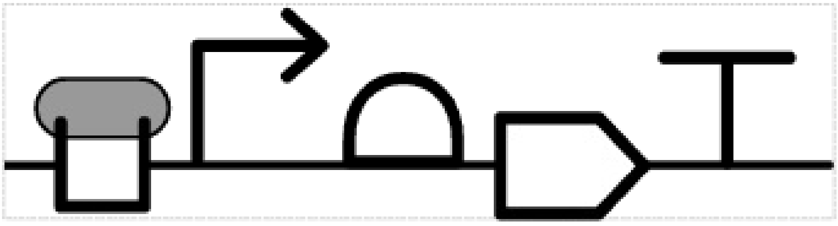

An alternative is the SBGN "cornered rectangle" glyph for a complex:

### Prototypical Example

Arabinose bound to AraC

### Notes

*this section deliberately blank*

## Double-Stranded Nucleic Acid

### Associated SBO term(s)

SBO:0000251 Deoxyribonucleic acid

### Recommended Glyph and Alternates

The RECOMMENDED glyph for dsNA is a double-helix:

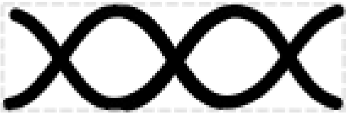

An alternative is the SBGN "nucleic acid" half-round rectangle:

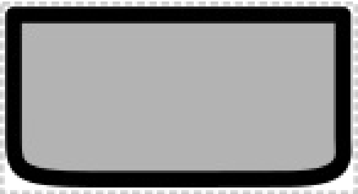

### Prototypical Example

DNA fragment during assembly

### Notes

*this section deliberately blank*

## Macromolecule

### Associated SBO term(s)

SBO:0000245 Macromolecule

### Recommended Glyph and Alternates

The macromolecule glyph is a rounded rectangle, as used in SBGN:

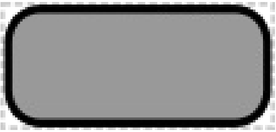

### Prototypical Example

AraC protein, polymerized chitin

### Notes

The alternative "shmoo" shape from SBOL Visual 2 is no longer valid, having been deprecated for looking too similar to a typical representation of a yeast cell.

## No Glyph Assigned

### Associated SBO term(s)

Any SBO type that is not covered by any glyph besides the root

### Recommended Glyph and Alternates

When a species has no assigned glyph it is RECOMMENDED that a user provide their own glyph. The user is also encouraged to submit the new glyph for possible adoption into the SBOLv standard.

An alternative option is to have a bracket, suggesting information that needs to be filled in:

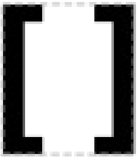

### Prototypical Example

No Glyph Assigned is intended to be used for any chemical species whose type is not covered by other SBOL Visual glyphs.

### Notes

No Glyph Assigned is intended for molecular species with a defined specific type that happens to not yet be covered by available approved glyphs (other than the root). It is more likely to appear in machine-generated diagrams than in human-generated diagrams, since humans are likely to invent and use their own glyph for the purpose.

## Protein

### Associated SBO term(s)

SBO:0000252 Polypeptide Chain

### Recommended Glyph and Alternates

The protein glyph is a "pill" shape with a rectangular body and rounded ends, representing the compact space-filling mass of many proteins:

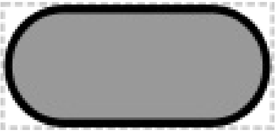

### Prototypical Example

AraC protein

### Notes

To avoid confusion with circles or ellipses, the "pill" shape SHOULD be significantly longer than it is tall, emphasizing its straight sides.

## Simple Chemical

### Associated SBO term(s)

SBO:0000247 Simple chemical

### Recommended Glyph and Alternates

The simple chemical glyph is any one of three small polygonal shapes, triangle, pentagon, or hexagon (in order: triangle, pentagon, hexagon):

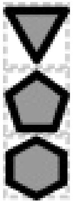

Alternately, a simple chemical may also be represented a small circle:

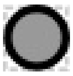

### Prototypical Example

Arabinose

### Notes

It is RECOMMENDED that visual differentiation be maximized by associating each distinct species in a diagram with a different small geometric shape. Rotations may also be used (e.g., pentagon pointing up vs. pentagon pointing down).

It is RECOMMENDED that labels should be placed outside of the shapes rather than inside, to avoid squeezing the labels.

To avoid confusion with pills or ellipses, when the small circle alternative glyph is used, it SHOULD be significantly smaller than other types of molecular species glyphs, as indicated by the recommended scale of the glyph.

## Single-Stranded Nucleic Acid

### Associated SBO term(s)

SBO:0000250 Ribonucleic acid

### Recommended Glyph and Alternates

The RECOMMENDED glyph for ssNA is a wiggly line:

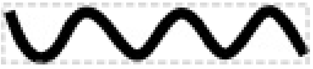

An alternative is the SBGN "nucleic acid" half-round rectangle:

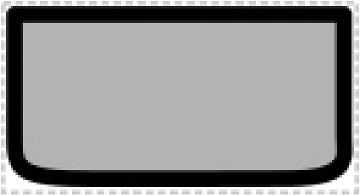

### Prototypical Example mRNA, gRNA, siRNA

### Notes

*this section deliberately blank*

## Unspecified

### Associated SBO term(s)

SBO:0000285 Material entity of unspecified nature

### Recommended Glyph and Alternates

Unspecified is RECOMMENDED to be represented by the unicode "replacement character" glyph, indicating a missing or invalid symbol:

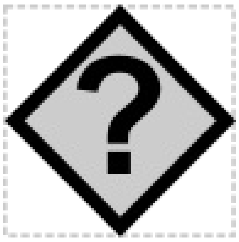

An alternative is the SBGN "generic species" glyph, which is an ellipse:

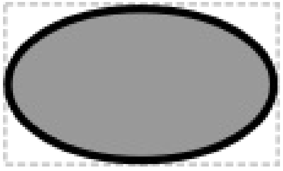

### Prototypical Example

An anonymous chemical species that is missing any information about its nature or intended purpose.

### Notes

The Unspecified glyph is intended for showing where a chemical species' type is missing (or, equivalently, given only the uninformative root role). It should never appear with well-curated designs or diagrams.

### A.3 Interaction Glyphs

These glyphs are different forms of "arrow" representing interactions between sequence features and/or molecular species. As arrows, they are extensible and do not have a separately identified bounding box.

## Control

### Associated SBO term(s)

SBO:0000168 Control

Head: SBO:0000644 Modified

Tail: SBO:0000019 Modifier

### Recommended Glyph and Alternates

An arrow with a diamond head:

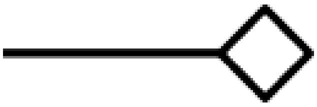

### Prototypical Example

Inversion of a sequence flanked by FRT sites by FLP recombinase

### Notes

*This section left intentionally blank*

## Degradation

### Associated SBO term(s)

SBO:0000179 Degradation

Head: n/a

Tail: SBO:0000010 Reactant

### Recommended Glyph and Alternates

Identical to the Process glyph, but with an empty set at the sink of the arrowhead:

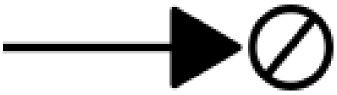

### Prototypical Example

Cellular recycling of mRNA

### Notes

*This section left intentionally blank*

## Inhibition

### Associated SBO term(s)

SBO:0000169 Inhibition

Head: SBO:0000642 Inhibited

Tail: SBO:0000020 Inhibitor

### Recommended Glyph and Alternates

An arrow whose head is a bar, suggesting blocking:

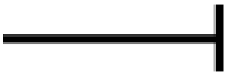

### Prototypical Example

Repression of pTAL14 promoter by TAL14

### Notes

*This section left intentionally blank*

## Process 1

### Associated SBO term(s)

SBO:0000375 Process

Head: SBO:0000011 Product

Tail: SBO:0000010 Reactant, SBO:0000645 Template (Genetic Production only)

### Recommended Glyph and Alternates

An arrow with a filled head the same color as the line:

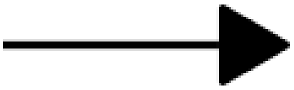

### Prototypical Example

Production of Green Fluorescent Protein (GFP) from the gfp Coding Sequence

### Notes

The assocated SBO term also covers:

- SBO:0000176 Biochemical Reaction
- SBO:0000589 Genetic Production (source is DNA, sink is usually RNA or Macromolecule)
- SBO:0000177 Non-covalent Binding (sink is a Complex)

## Stimulation

### Associated SBO term(s)

SBO:0000170 Stimulation

Head: SBO:0000643 Stimulated

Tail: SBO:0000459 Stimulator

### Recommended Glyph and Alternates

An arrow with an head that is empty or of a different color than the line:

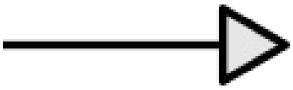

### Prototypical Example

Activation of pTAL14 promoter by Gal4VP16 activator

### Notes

*This section left intentionally blank*

### A.4 Interaction Node Glyphs

These glyphs are placed at the junctions of edges to represent biochemical processes, and include a bounding box (grey dashed box) but are not connected to any nucleic acid backbone. Grey dashed lines provide examples of how edges may connect to the glyph.

## Association

### Associated SBO term(s)

SBO:0000177 Non-Covalent Binding

Incoming: SBO:0000010 Reactant

Outgoing: SBO:0000011 Product

### Recommended Glyph and Alternates

A circular node:

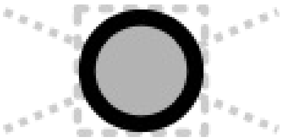

### Prototypical Example

Association of gRNA and Cas9 to form an active CRISPR complex.

### Notes

The association glyph is based on the SBGN Process Description association glyph.

## Dissociation

### Associated SBO term(s)

SBO:0000180 Dissociation

Incoming: SBO:0000010 Reactant

Outgoing: SBO:0000011 Product

### Recommended Glyph and Alternates

An circular node inside another circle

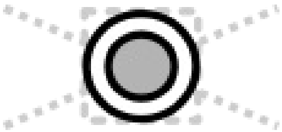

### Prototypical Example

Dissociation of an active CRISPR complex into gRNA and Cas9.

### Notes

The dissociation glyph is based on the SBGN Process Description dissociation glyph.

## Process

1

### Associated SBO term(s)

SBO:0000375 Process

Incoming: SBO:0000010 Reactant

Outgoing: SBO:0000011 Product

### Recommended Glyph and Alternates

A square node:

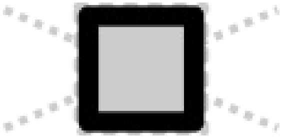

### Prototypical Example

Reaction process of citrate and aconitase to produce cis-aconitate.

### Notes

The process glyph is based on the SBGN Process Description process glyph.

The assocated SBO term also covers:

- SBO:0000176 Biochemical Reaction
- SBO:0000177 Non-covalent Binding (sink is a Complex)

Examples of RECOMMENDED usage:

- Biochemical reaction process of the reactant citrate and the catalyst aconitase to produce the product cis-aconitate:

1

## Unspecified 1

### Unspecified SBO term(s)

SBO:0000231 occurring entity representation

Incoming: SBO:0000003 Participant Role

### Recommended Glyph and Alternates

Unspecified is represented by the unicode "replacement character" glyph, indicating a missing or invalid symbol:

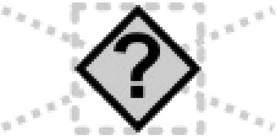

### Prototypical Example

An interaction that is missing any information about its nature or intended purpose.

### Notes

Note that there are no outgoing edges for the Unspecified interaction, because there is no difference in roles to indicate.

The Unspecified glyph is intended for showing where information about an interaction is missing. It should not generally appear with well-curated designs or diagrams.

## B Examples

This section contains prototypical examples, including use of all current glyphs to attempt to ensure that their use is clear.

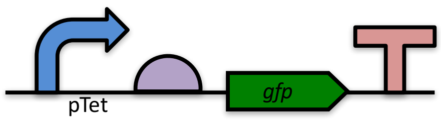

***Figure 27:*** *DNA sequence for a functional unit in which the pTet promoter and an anonymous ribosome entry site regulate expression of a coding sequence for GFP, ended by a terminator.*

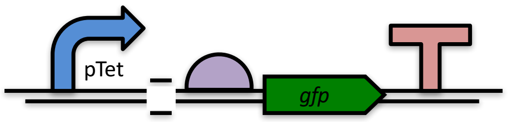

***Figure 28:*** *The same functional unit as in the previous figure, with additional assembly-focused information: there is a 5' overhang before the promoter, a 3' overhand after the terminator, and an assembly scar between the promoter and the ribosome entry site left over from a prior step of assembly.*

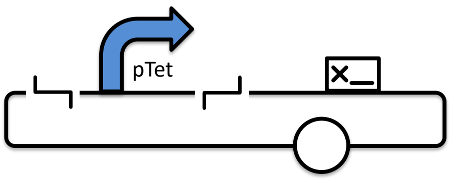

***Figure 29:*** *Promoter pTet stored in a circular plasmid. The promoter is prepared for being cut out of the plasmid: it is preceded by a 5' sticky end restriction site and followed by a 3' stick end restriction site. In addition, the plasmid has been bar-coded with a signature and has its origin of replication marked.*

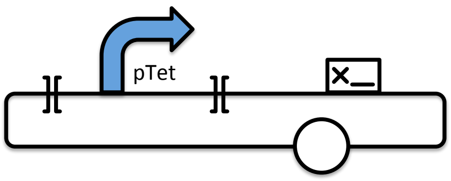

***Figure 30:*** *Promoter pTet stored in a circular plasmid, flanked by blunt-ended restriction sites. In addition, the plasmid has been bar-coded with a signature and has its origin of replication marked.*

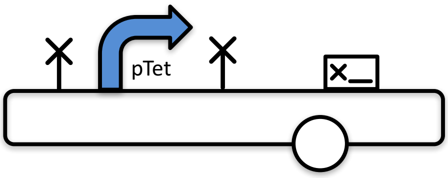

***Figure 31:*** *Promoter pTet stored in a circular plasmid, flanked by restriction sites with unspecified cut structure. In addition, the plasmid has been bar-coded with a signature and has its origin of replication marked.*

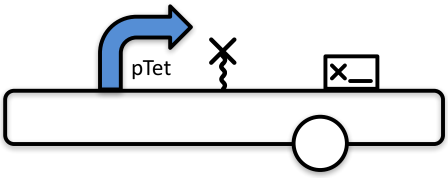

***Figure 32:*** *Circular plasmid containing a pTet promoter followed by a ribonuclease site. In addition, the plasmid has been bar-coded with a signature and has its origin of replication marked.*

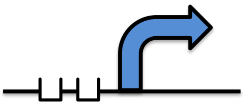

***Figure 33:*** *Detailed design of a promoter, in which the transcription start site is preceded by two operator sites where regulators bind.*

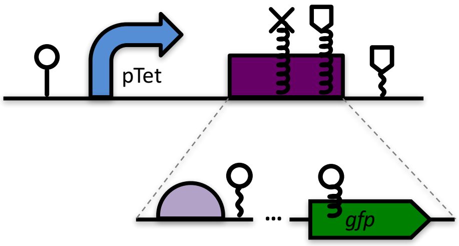

***Figure 34:*** *Promoter regulating the production of an engineered composite sequence that includes RNA and protein stability elements at its 3' end, as well as an internal site for protease cleavage, as well as the expansion of the composite to show it contains a ribosome entry site. coding sequence, and other omitted details. Single residue locations of interest are indicated for the DNA (before the promoter), RNA (after the ribosome entry site), and protein (in the CDS).*

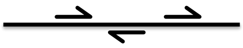

***Figure 35:*** *DNA sequence with three primer binding sites.*

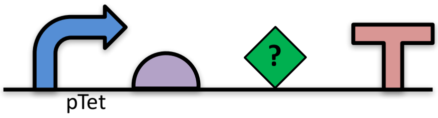

***Figure 36:*** *A functional unit consisting of promoter, ribosome entry site, CDS, and terminator, except that information about the CDS is missing, leaving it to fall back on the default unspecified glyph.*

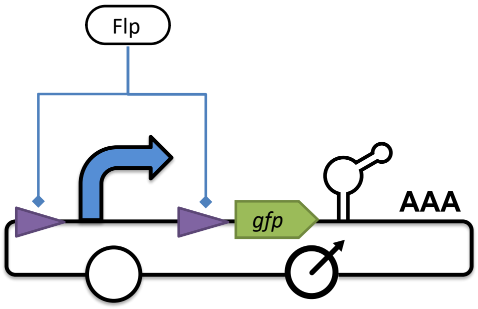

***Figure 37:*** *Promoter regulating the expression of GFP, which is also regulated by an aptamer between it and the poly-A tail of the transcript. The promoter can be cut out by a pair of recombinase target sites, which are acted on by the Flp protein. The whole construct is stored in a circular plasmid with an origin of replication and also an origin of transfer.*

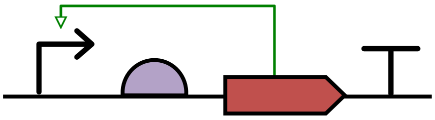

***Figure 38:*** *Promoter stimulated by the CDS that it regulates.*

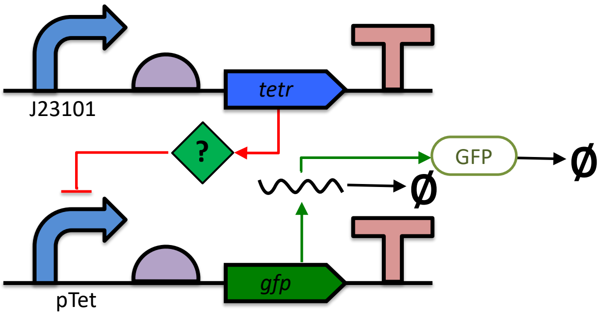

***Figure 39:*** *Constitutive production of TetR, except that information about the protein is missing, leaving it as the default unspecified glyph. TetR represses the pTet promoter, which is regulating production of GFP. The diagram of GFP production explicitly includes the intermediate mRNA and the degradation of both the mRNA and protein products.*

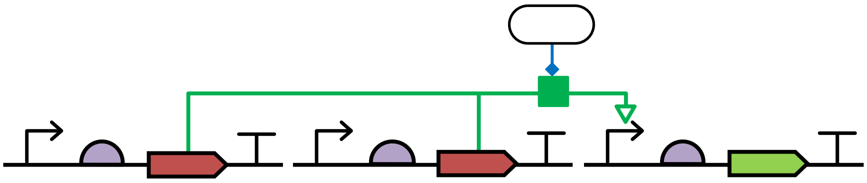

***Figure 40:*** *Phosphorylation of an inactive transcription factor (produced by two different CDSs) by a kinase to form an active transcriptional activator, which then stimulates a promoter.*

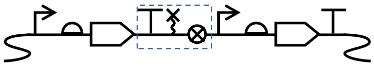

***Figure 41:*** *Ribozyme and spacer between two transcriptional units, forming a separation module with the preceding terminator (dashed box), with the whole construct integrated into the chromosome.*

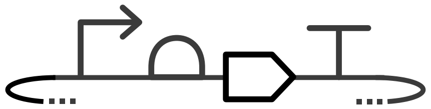

***Figure 42:*** *A circular plasmid containing a functional unit consisting of promoter, ribosome entry site, CDS, and terminator.*

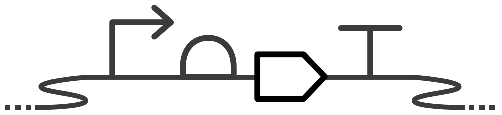

***Figure 43:*** *A functional unit consisting of promoter, ribosome entry site, CDS, and terminator, all integrated together into the chromosome.*

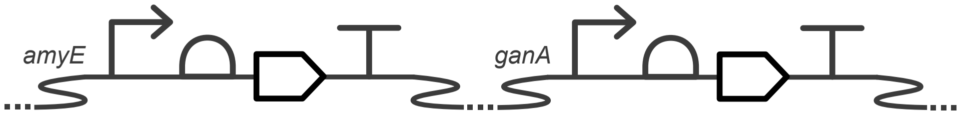

***Figure 44:*** *Two functional units, one integrated into the amyE locus, another integrated into the ganA locus.*

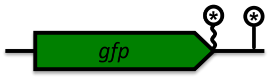

***Figure 45:*** *Coding sequence marking the stop codon at its end and transcriptional end site farther down the sequence.*

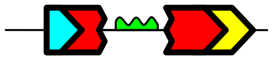

***Figure 46:*** *A coding sequence with three domains: an N-tag (blue), C-tag (yellow), and internal region (red) interrupted by an intron that includes a gRNA non-coding RNA sequence (green).*

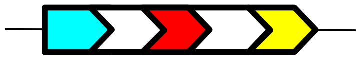

***Figure 47:*** *A coding sequence with three designated domains, an N-tag (blue), C-tag (yellow), and internal region (red).*

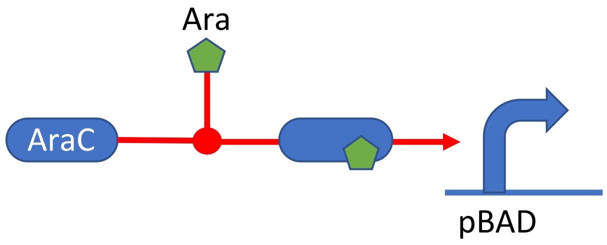

***Figure 48:*** *The AraC protein and arabinose associating into a complex that activates the pBAD promoter.*

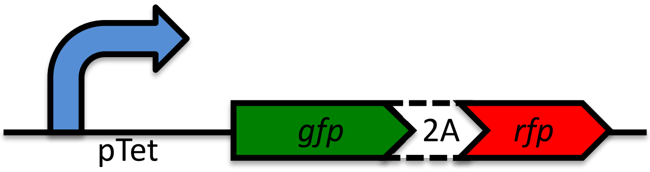

***Figure 49:*** *Bicistronic expression of both GFP and RFP from a signal coding sequence, by means of a 2A self-cleaving polypeptide region.*

## C Mapping between to SBOL Visual 1, 2, 3

### C.1 Mapping between SBOL Visual 1 and SBOL Visual 2

SBOL Visual 2 differs from SBOL Visual 1 in the following major ways:

- Diagram syntax is expanded to include functional interactions and molecular species.
- The relationship between diagrams and the SBOL data model is made explicit.
- A number of requirements and best practices are specified for glyphs and diagrams, including:
  - Glyphs include information on interior, bounding box, and recommended backbone alignment.
  - Sequence feature glyphs are required to have their bounding boxes contact the nucleic acid backbone.
  - Nucleic acid diagrams now require the nucleic acid backbone line, and the number of lines allowed in various circumstances is constrained.
  - Explicit statement of when a glyph can and cannot be used to represent a particular element of a diagram.
- Labels that name objects are distinguished from other types of textual annotation.
- Explicit statement of which aspects of a symbol are *not* controlled.
- Symbol variants are now supported.

In addition, the collection of sequence feature glyphs have been expanded and modified in the following ways:

- All non-ambiguous glyphs have been provided with bounding box, interior, and recommended backbone alignment.
- The User Defined glyph has been split into Unspecified, No Glyph Assigned, Engineered Region, and Composite.
- Glyphs have been added for Aptamer, Biopolymer Location, Chromosomal Locus, Circular Plasmid, Inert DNA Spacer, Intron, Non-Coding RNA Gene, Omitted Detail, Origin of Transfer, PolyA Site, Polypeptide Region, Specific Recombination Site, and Stop Site.
- The following ontology terms have been assigned or adjusted:
  - Ribonuclease Site has been assigned SO:0001977.
  - 5’ Sticky End Restriction Site has been assigned SO:0001975.
  - 3’ Sticky End Restriction Site has been assigned SO:0001976.
  - Signature has been assigned SO:0001978.
  - RNA Stability Element has been updated from the obsolete SO:0001957 to the current SO:0001979
  - Restriction Enzyme Recognition Site, in addition to SO:0000139 has a second definition as SO:0000061.
  - 5’ Overhang Site and 3’ Overhang Site were erroneously listed with their ontology terms exchanged; this has been fixed.

### C.2 Mapping between SBOL Visual 2 and SBOL Visual 3

The major difference between SBOL Visual 3 and SBOL Visual 2 is that diagrams and glyphs are defined with respect to the SBOL 3 data model rather than the SBOL 2 data model. SBOL Visual 3 diagrams may still be related to the SBOL 2 data model by following the mapping between SBOL 3 and SBOL 2 data models provided in the SBOL 3 specification.

A byproduct of this change is that the use of dashed undirected lines for subsystem mappings has been removed. In SBOL Visual 2, dashed line mappings were analogous to an SBOL 2 `MapsTo`, which is a compound mapping relationship indicating both reference into a subsystem and one of several identity relationships. In SBOL 3, these functions have been divided between two classes, `Constraint` to indicate relationships (including identity) and `ComponentReference` to access subsystem features. In SBOL Visual 3, interactions crossing a subsystem boundary line indicate access of subsystem features via `ComponentReference`. As SBOL 3 `Constraint` objects can express many other relationships besides identity, however, the potential use of dashed undirected lines to indicate identity relationships is currently reserved as a potential future addition to the SBOL Visual 3 specification, but not yet implemented. Until a decision is made about how to represent these relationships, the specification is mute on both constraints and dashed undirected lines, which means that it is acceptable to use them, if desired, as an annotation indicating identity.

In addition, collection of glyphs has been modified as follows:

- The deprecated Insulator glyph and “shmoo” Macromolecule alternative glyph have been removed.
- Deprecated BioPAX alternatives to SBO terms for molecular species glyphs have been removed.
